# H3F3A K27M Mutation Promotes the Infiltrative Growth of High-Grade Glioma in Adults by Activating β-Catenin/USP1 Signaling

**DOI:** 10.3390/cancers14194836

**Published:** 2022-10-03

**Authors:** Zhiyuan Sun, Yufu Zhu, Xia Feng, Xiaoyun Liu, Kunlin Zhou, Qing Wang, Hengzhu Zhang, Hengliang Shi

**Affiliations:** 1 Central Laboratory, The Affiliated Hospital of Xuzhou Medical University, Xuzhou 221002, China; 2 Institute of Digestive Diseases, Xuzhou Medical University, Xuzhou 221002, China; 3 Department of Neurosurgery, The Affiliated Hospital of Xuzhou Medical University, Xuzhou 221002, China; 4 Department of Neurosurgery, The Affiliated Wuxi Second Hospital, Nanjing Medical University, Wuxi 214002, China; 5 Department of Neurosurgery, Clinical Medical College, Yangzhou University, Yangzhou 225009, China

**Keywords:** H3F3A, H3.3K27M, glioma, infiltration

## Abstract

**Simple Summary:**

Gliomas is a primary type of tumor in the central nervous system. High-grade glioma is a malignant cancerous disease and grows rapidly. This study reports the expression of H3.3K27M in high-grade glioma tissues and the association with malignant glioma cell behavior. Moreover, the results suggested that a high expression of H3.3K27M promotes the migration and invasion of glioma cells, leading to a poor prognosis by promoting the infiltration of glioma through aggravating aberrant activation of β-catenin signaling-driven pathway.

**Abstract:**

H3F3A K27M (H3.3K27M) is a newly identified molecular pathological marker in glioma and is strongly correlated with the malignancy of diffuse intrinsic pontine glioma (DIPG). In recent years, accumulating evidence has revealed that other types of glioma also contain the H3.3K27M mutation. However, the role of H3.3K27M in high-grade adult glioma, the most malignant glioma, has not been investigated. In this study, we focused on exploring the expression and function of H3.3K27M in high-grade glioma in adults. We found that H3.3K27M was highly expressed at high levels in some high-grade glioma tissues. Then, we introduced H3.3K27M into H3.3 wild-type glioma cells, U87 cells and LN229 cells. We found that H3.3K27M did not affect the growth of glioma cells in vitro and in vivo; however, the survival of mice with transplanted tumors was significantly reduced. Further investigation revealed that H3.3K27M expression mainly promoted the migration and invasion of glioma cells. Moreover, we confirmed that H3.3K27M overexpression increased the levels of the β-catenin and p-β-catenin (Ser675) proteins, the ubiquitin-specific protease 1 (USP1) mRNA and protein levels, and the enhancer of zeste homolog 2 (EZH2) protein level. In addition, the β-catenin inhibitor XAV-939 significantly attenuated the upregulation of the aforementioned proteins and inhibited the increased migration and invasion caused by the H3.3K27M mutation. Overall, the H3.3K27M mutation in high-grade glioma is a potential biomarker for poor prognosis mainly due to the infiltration of glioma cells that is at least partially mediated by the β-catenin/USP1/EZH2 pathway.

## 1. Introduction

Gliomas are a primary type of tumor in the central nervous system. In the 2016 World Health Organization (WHO) classification, glioblastoma (GBM) was defined as a grade IV glioma and comprises 45.2% of all malignant central nervous system (CNS) tumors and 80% of all primary malignant CNS tumors [1,2]. GBMs and other high-grade gliomas are severe diseases, as no effective treatment exists. Surgical treatment, radiotherapy and chemotherapy are the main treatment methods, and the prognosis is poor, despite the addition of a second surgery and re-irradiation [3,4,5]. Therefore, many neuroscientists have expended increasing effort into studying malignant glioma to identify effective ways to cure this disease.

Histones are the core components of the nucleosome subunit; they form an octamer containing four core histones (H3, H4, H2A, and H2B) surrounded by a 147-base pair DNA fragment. Histone tails are influenced by a wide range of covalent posttranslational modifications (PTMs) that jointly regulate the chromatin status [6]. These PTMs change the electronic charge and structures of these histone tails, which bind to the DNA, to alter the chromatin status and subsequent gene expression [7]. The PTM of histones is closely related to the occurrence of many tumors [8,9]. As histone mutations directly affect histone PTMs that are associated with gene activity, these mutations likely contribute to tumor development through either the activation of oncogenes or the repression of essential tumor suppressor genes [10]. Thus, the role of the H3.3 mutation in the mechanism of tumor development must be explored.

Schwartzentruber et al. first reported that the K27M mutation exists in pediatric glioblastoma [11]. In the 2016 WHO classification, the H3 K27M mutant was first added to the list of diffuse gliomas as a new classification [1]. In recent years, scientists have focused on H3.3K27M-mutant diffuse midline gliomas. However, an increasing number of scholars have reported that some other types of intracranial tumors, such as ependymomas and gangliogliomas, exhibit the H3.3K27M mutation [12,13]. Researchers found that K27M-mutant diffuse midline gliomas are associated with significantly shorter survival among patients with all midline tumor locations [14]. Accordingly, H3.3K27M plays an important role in the occurrence and progression of intracranial tumors. Nevertheless, the potential molecular mechanisms of H3.3K27M in other types of glioma are not very clear and require further investigation.

Enhancer of zeste homolog 2 (EZH2) is a significant constituent of polycomb repressive complex 2 (PRC2). It functions as an oncogene in the occurrence and progression of tumors by regulating epigenetic genes [15]. PRC2 is a chromatin-associated methyltransferase catalyzing the mono-, di-, and trimethylation of lysine 27 on histone H3 (H3K27) [16]. EZH2 has been reported as a potential therapeutic target for H3K27M-mutant pediatric gliomas [17]. However, the function of EZH2 in adults with H3K27M-mutant glioma is not very clear.

In this study, we first assessed the expression of H3.3K27M in patients with glioma and glioma cell lines using western blotting. Then, we introduced the H3.3K27M mutation into glioma cells and investigated its effect on proliferation, migration and invasion. Finally, we investigated the underlying mechanisms by which the H3.3K27M mutation promotes glioma progression.

## 2. Materials and Methods

### 2.1. Glioma Specimens

Glioma tissues and normal brain tissues were collected at the Affiliated Hospital of Xuzhou Medical University (Xuzhou, Jiangsu, China). All human glioma specimens were assessed to confirm the pathological diagnosis. The research was approved by the Research Ethics Committee of Xuzhou Medical University, and written informed consent was obtained from patients who underwent surgery at the Affiliated Hospital of Xuzhou Medical University.

### 2.2. Antibodies and Reagents

Antibodies specific for AKT (#4685), P-AKT (Ser473, #4060), MEK1/2 (#4694), P-MEK1/2 (Ser217/221, #9154), STAT3 (#12640), P-STAT3 (#9145), β-catenin (#8480), P-β-catenin (Ser675, #4176), H3K27M (#74829), H3 (#4499), H3K27me3 (#9733), Myc-Tag (#2276), GAPDH (#5174) and β-actin (#8457) were purchased from Cell Signaling Technology (Danvers, MA, USA). USP1 (14346-1-AP), EZH2 (21800-1-AP) and PKA c-beta (55382-1-AP) antibodies were purchased from Proteintech (Wuhan, China). The dilutions of antibodies from Cell Signaling Technology were 1:1000 and the antibodies from Proteintech were diluted 1:2000. XAV-939, H89 and puromycin were obtained from MedChemExpress (Shanghai, China).

### 2.3. Cell Culture

All of the cell lines used in the experiment (HEK-293T, U251, U87, LN229, U118, A172 and T98G) were purchased from Shanghai Cell Bank, Chinese Academy of Sciences. Cells were cultured in Dulbecco’s modified Eagle’s medium (DMEM) (Gibco, CA, USA) containing 10% fetal bovine serum (Invitrogen, Carlsbad, CA, USA) and incubated in an incubator at 37 °C with 5% CO_2_.

### 2.4. Western Blotting

The cells were collected on ice and lysed in RIPA buffer (50 mM Tris (pH 7.4), 150 mM NaCl, 1% Triton X-100, 1% sodium deoxycholate, 0.1% SDS, and 1% NP-40). Proteins were harvested after centrifugation at 12,000 rpm. Equal amounts of samples were separated on 8%, 10% or 12% SDS-PAGE gels and then transferred to 0.22 μm PVDF membranes (Roche Diagnostics GmbH, Mannheim, Germany). The membranes were blocked with 3% bovine serum albumin (BSA) for 2 h and then incubated overnight at 4 °C with diluted primary antibodies (AKT, P-AKT, MEK1/2, P-MEK1/2, STAT3, P-STAT3, β-catenin, P-β-catenin (Ser675), USP1, EZH2, H3K27M, H3, H3K27me3, Myc-Tag, GAPDH, and β-actin). The next day, the membranes were incubated with the secondary antibody for 2 h. Finally, the membranes were detected using an enhanced chemiluminescence detection system (Thermo Fisher, Waltham, MA, USA).

### 2.5. RNA Isolation, cDNA Synthesis and RT-PCR

RNA was extracted from stable cell lines by using TRIzol (Beyotime, Shanghai, China), and cDNAs were synthesized by employing a Prime Script RT Reagent Kit (TIANGEN, Beijing, China) according to the manufacturer’s instructions. The target genes were amplified in a final volume of 20 μL with SYBR Green PCR Master mix (TIANGEN, Beijing, China). Quantitative RT-PCR was performed using an ABI7300 real-time PCR instrument (Applied Biosystems, Carlsbad, CA, USA) with SYBR Green. The primers used to amplify USP1 and β-actin were as follows: USP1-F: 5-GCT GCT AGT GGT TTG GAG TTT-3, USP1-R: 5-GCA TCA CAA CCG CAA ATA ATC C-3; β-Actin-F: 5-CCA ACC GCG AGA AGA TGA-3 and β-Actin-R: 5-CCA GAG GCG TAC AGG GAT AG-3.

### 2.6. H3F3A Gene Sequencing

The cDNAs from the samples were synthesized using the aforementioned methods, and then the H3F3A gene was amplified in a final volume of 20 μL with a 2 × Taq PCR Mix Kit (TIANGEN, Beijing, China). The final products were sent to the Genscript Biotechnology Company (Nanjing, China) for gene sequencing. The following primers were used for the amplification and sequencing of H3F3A were as follows: H3F3A-F: 5-ATG GCT CGT ACA AAG CAG AC-3 and H3F3A-R: 5-AGC ACG TTC TCC ACG TAT GC-3.

### 2.7. Lentivirus Packaging and Stable Cell Lines

The wild-type Myc-tagged H3F3A or Myc-tagged H3.3K27M cDNA was subcloned into a lentiviral vector (pCDH-CMV-MCS-EF1-GFP, where the inserted gene and GFP were expressed independently. Lentiviruses were produced in HEK-293T cells by transfecting the core plasmid and two assistant plasmids (pMD2.G and psPAX2) with PolyJet reagent (SignaGen Laboratories, Frederick, MD, USA). We established stable cell lines by infecting U87 and LN229 cells with the vector, Myc-H3F3A or Myc-H3.3K27M viruses and screening them with puromycin (2.5 μg/mL) after 48 h of infection.

### 2.8. Cell Counting Kit-8 (CCK-8) Assay

Four thousand cells in 200 μL of medium were seeded in a 96-well plate. At the designated time points, the original medium was replaced with medium containing 10% CCK-8 reagent (Victimed, Xuzhou, China) and incubated at 37 °C for 2 h. Afterward, the absorbance at 450 nm was detected with a microplate reader. Cell viability was calculated based on the absorbance values.

### 2.9. Colony Formation Assay

Five hundred cells suspended in 5 mL of medium were inoculated into each 60 mm dish and cultured continuously until macroscopic cell colonies were formed. Then, the cells were fixed with 100% methanol and stained with a 0.1% crystal violet solution for 15 min. After washes with PBS, the colonies in the plates were photographed using a digital camera. Colonies containing more than 50 cells were counted manually.

### 2.10. Wound Healing Assay

Stable cell lines were inoculated in six-well plates and grown under normal conditions for 24 h. On the second day, the best cell density to perform the scratch assay was chosen when cells were close to confluent. After the scratch was generated with a pipette tip in the middle of the wells, the unattached cells were washed with PBS twice, and the culture medium was replaced with serum-free culture medium. Photographs were captured at 0 h, 24 h, 48 h or 0 h, 12 h and 24 h using an inverted microscope (IX71; Olympus, Tokyo, Japan).

### 2.11. Transwell Invasion and Migration Assays

Transwell assays were performed with a polycarbonate filter membrane with a diameter of 6.5 mm and pore size of 8 μm (Corning, Bedford, MA, USA) according to the manufacturer’s protocol. Matrigel (BD, San Jose, CA, USA) was used to precoat the filters to analyze cell invasion. Trypsin-treated cells were resuspended in serum-free medium. A total of 100 μL of the cell suspension containing 3000 cells was added to the upper compartment, and 500 μL of 3% FBS medium were added to the lower compartment as a chemoattractant. After an incubation at 37 °C for 36 h, the chamber was washed twice with PBS to remove the noninvasive cells from the upper surface. Then, the filters were fixed with methanol for 30 min. After two washes with PBS, the noninvasive cells were gently removed from the upper surface with a cotton swab, and crystal violet was added and incubated with the cells for 30 min. After drying, photographs of five randomly selected fields from each well were captured using an inverted microscope. The same experimental design was used for migration experiments except that the filters were not pretreated with Matrigel.

### 2.12. Orthotopic Mouse Model and In Vivo Imaging Analysis in Nude Mice

Animal experiments were approved by the ethics committee and met the standards required by the guidelines of Xuzhou Medical University (Xuzhou, China). First, we constructed luciferase-mCherry-U87 cells, and then we used lentiviruses to construct cells that are stably expressed the vectors Myc-H3F3A and Myc-H3.3K27M. After anesthetizing all the nude mice, we incised the skin of the mouse head to a size of approximately 1 cm and then drilled a small hole 1.8 mm right lateral to bregma. Then, the constructed U87 cells (5 × 10^5^) were diluted in L15 medium and injected at 3 mm below the dura of 7-week-old male nude mice by a microinjector. Finally, the incision was sutured, and all mice were rewarmed and returned to the cage. On the seventh day after transplantation, the luciferase fluorescence intensity in the nude mice was observed using the IVIS kinetic imaging system. Briefly, the nude mice were injected with a fluorescein potassium solution, and the luciferase intensity in the nude mice was observed using the IVIS kinetic imaging system. The glioma growth rate was assessed by analyzing the luciferase intensity in the nude mice. Mice were sacrificed when they exhibited hemiplegia, listlessness, cachexia and other neurological symptoms to obtain the survival curve.

### 2.13. Hematoxylin and Eosin Staining

For hematoxylin and eosin (HE) staining, sections were dewaxed in xylene, hydrated with a gradient of alcohol solutions and then rinsed with tap water. The sections were sequentially stained with a hematoxylin and eosin dye solution for 5 min each, dehydrated and sealed with neutral resin. The sections were observed and photographed under a microscope.

### 2.14. Statistical Analysis

All experiments were performed three times, and the data were presented as the mean ± SD. Data were analyzed with GraphPad Prism 7 sofware. One-way ANOVA, two-way ANOVA and Tukey’s multiple comparisons test were used to analyze differences in each three-group comparison. Overall survival curves were generated using the Kaplan–Meier method and compared using the log-rank test. *p* < 0.05 was considered to indicate statistical significance.

## 3. Results

### 3.1. The H3.3K27M Mutation Exists in Human Patients with Glioma

**Figure 1:**
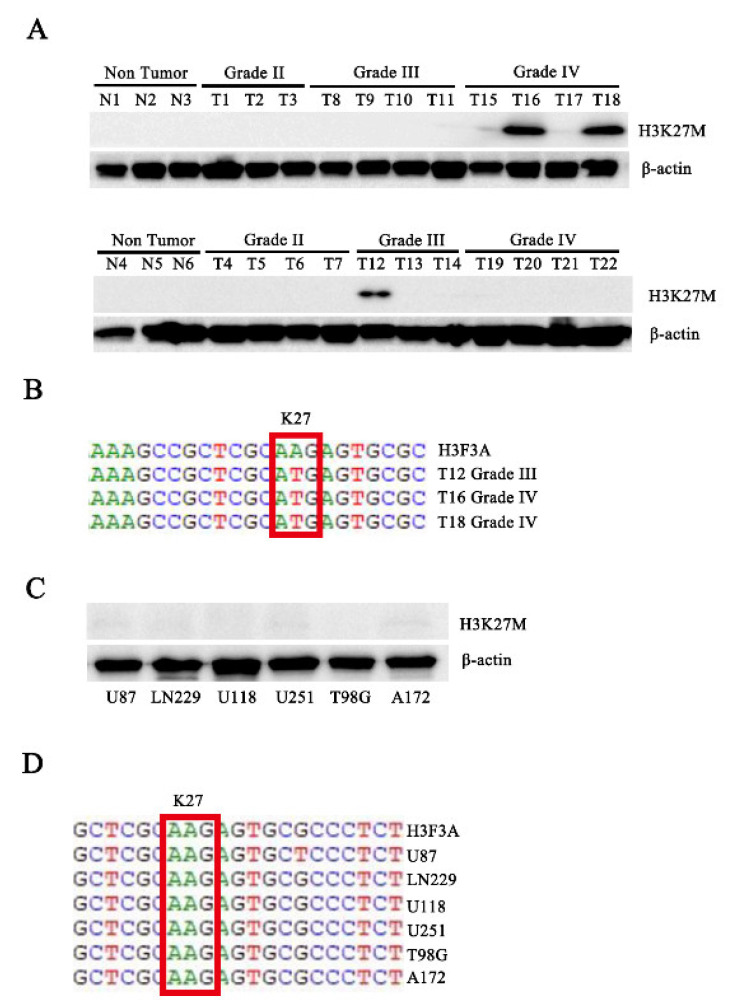
Western blotting and sequencing confirmed that the H3.3K27M mutation exists in human patients with glioma. (**A**) Western blot analysis of total proteins isolated from cancer tissue specimens of 28 to assess H3K27M protein levels. N indicates nontumor tissue, and T indicates tumor tissue. (**B**) Gene sequence showing the H3.3K27M mutation in three samples. (**C**) Six glioma cell lines were used to assess H3K27M protein levels using Western blotting. (**D**) Gene sequencing of the H3.3K27M mutation in six glioma cell lines.

We performed Western blot assays with the total protein from six nontumor tissues and 22 glioma tissues (Supplementary Table S1) using the H3K27M antibody to assess the expression of H3.3K27M in patients with glioma. Interestingly, the H3K27M mutation was detected in three patients with glioma, all of whom were diagnosed with high-grade glioma (one sample was grade III, and two samples were grade IV) (Figure 1A). Subsequently, we performed DNA sequencing to confirm the mutation in these three tissues (Figure 1B). All of these sequences mutated from the AAG codon to the ATG codon at position 27. Finally, to confirm whether the mutation was present in the commonly used glioma cell lines, we assessed the H3.3K27M mutation status in six glioma cell lines (U87, LN229, U118, U251, T98G, and A172). However, the H3.3K27M mutation was not detected in these cell lines (Figure 1C,D). 

### 3.2. H3.3K27M has no Obvious Effect on Glioma Cell Proliferation but Promotes Glioma Cell Migration and Invasion In Vivo

**Figure 2:**
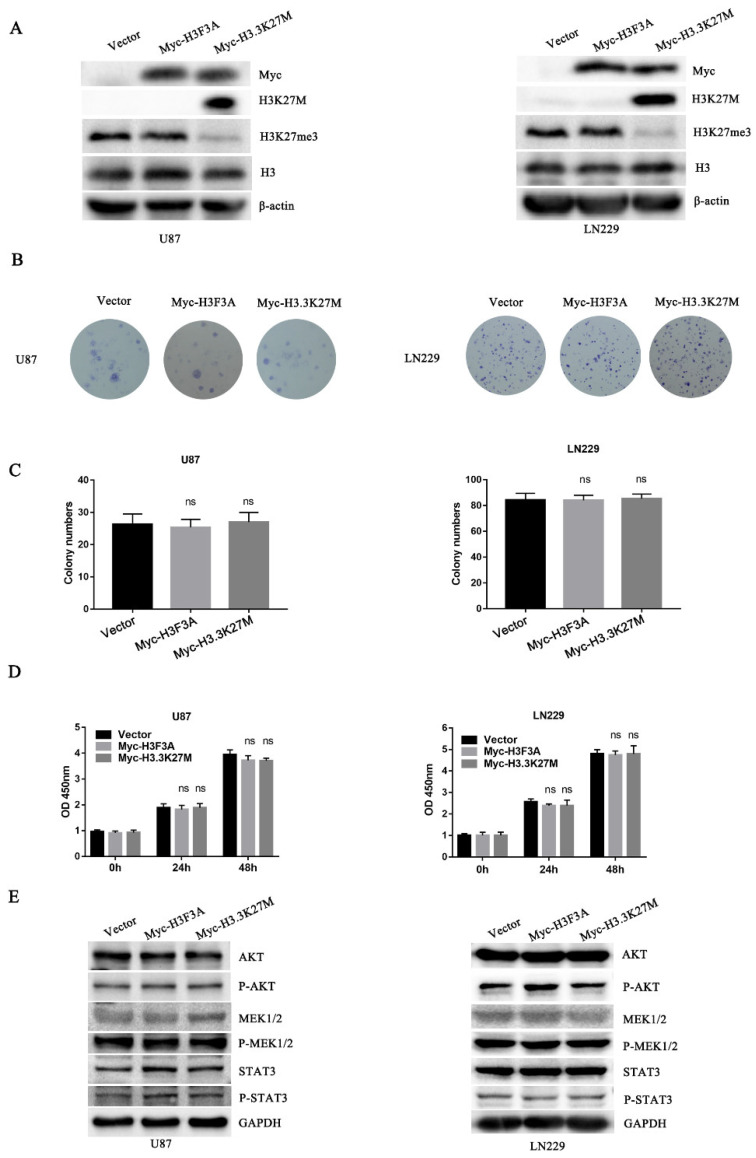
Colony formation and CCK-8 assays showed that H3.3K27M had no obvious effect on proliferation of glioma cells *in vitro*, and Western blotting showed that H3.3K27M did not change the levels of kinases involved classical proliferation pathways. (**A**) The overexpression efficiency in U87 and LN229 cells was verified using Western blotting. H3K27M was accompanied by a loss of H3K27me3, and no difference in the total amount of H3 was observed among the three groups. Representative images (**B**) and statistical analysis (**C**) of colony formation showed no discernible difference among the vector group, Myc-H3F3A group and Myc-H3.3K27M group in U87 and LN229 cells. (**D**) The CCK-8 assay revealed no significant effect on the proliferation of the three groups of U87 and LN229 cells. (**E**) Representative blots of AKT, P-AKT, MEK1/2, P-MEK1/2, STAT3 and P-STAT3 levels in the three groups of U87 and LN229 cells. ns indicates no statistical significance.

Due to the lack of H3.3K27M in these cell lines, we introduced the mutation by constructing cell lines stably expressing H3.3K27M. Additionally, we established a cell line overexpressing wild-type H3.3 as a negative control and a cell line overexpressing an empty vector as a blank control. The successful generation of these cell lines was confirmed by Western blotting (Figure 2A). After constructing the cell lines, we focused on exploring the role of H3.3K27M in tumorigenesis. Colony formation and CCK-8 assays were performed to determine the role of H3.3K27M in glioma cell proliferation. The expression of H3.3K27M expression had no effect on the proliferative ability of U87 and LN229 glioma cells (Figure 2B–D). Further detection at the molecular level indicated that H3.3K27M overexpression did not change the expression of proteins in three classical proliferation pathway-related kinases, including the AKT, MEK1/2 and STAT3 pathways (Figure 2E). 

**Figure 3:**
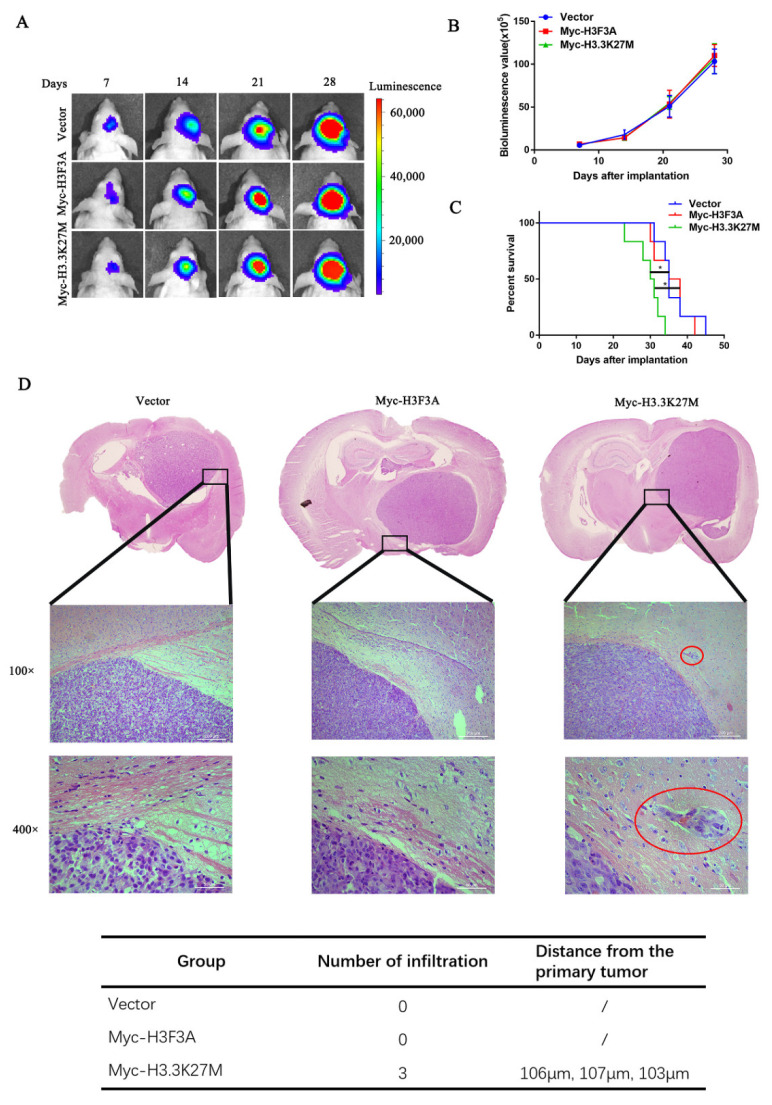
H3.3K27M overexpression had no effect on the proliferation of glioma cells but enhanced their invasion and migration *in vivo*. Bioluminescent imaging (**A**) and quantification analysis (**B**) of nude mice showed that H3.3K27M had no distinct effect on the growth rate of tumors compared with the vector group and Myc-H3F3A group. (**C**) Kaplan–Meier survival curve of nude mice injected orthotopically. H3.3K27M reduced the survival ability of nude mice. (**D**) Representative images of H&E staining in brain tissue. The Myc-H3.3K27M group showed greater infiltration than the other two groups. The red circle indicates the infiltration site. The table shows the number of infiltrating cells and their distance from the primary tumor. In this experiment, one mouse was randomly selected from each group on the 18th, 21st and 24th days after glioma cell inoculation for sectioning and H&E staining. In the vector and wild-type H3 groups, we did not observe tumor cell infiltration, but the infiltration of tumor cells were identified in the sections from three mice in the H3.3K27M mutant group. * *p* < 0.05; ns indicates no statistical significance.

Finally, we established a tumor model to confirm the above results described above by transplanting the aforementioned three cell lines expressing luciferase into the right striatum of nude mice. The tumor size was measured by performing the luciferase assay every seven days. H3.3K27M overexpression had no obvious effect on tumor growth (Figure 3A,B). However, H3.3K27M expression significantly reduced the survival rate of mice (Figure 3C). Moreover, by performing H&E staining of mouse brain slices, we found that a large number of invasive tumor cells infiltrated into the surrounding tissues from primary tumors in the H3.3K27M group (Figure 3D).

### 3.3. H3.3K27M Promotes Glioma Cell Migration and Invasion In Vitro

**Figure 4:**
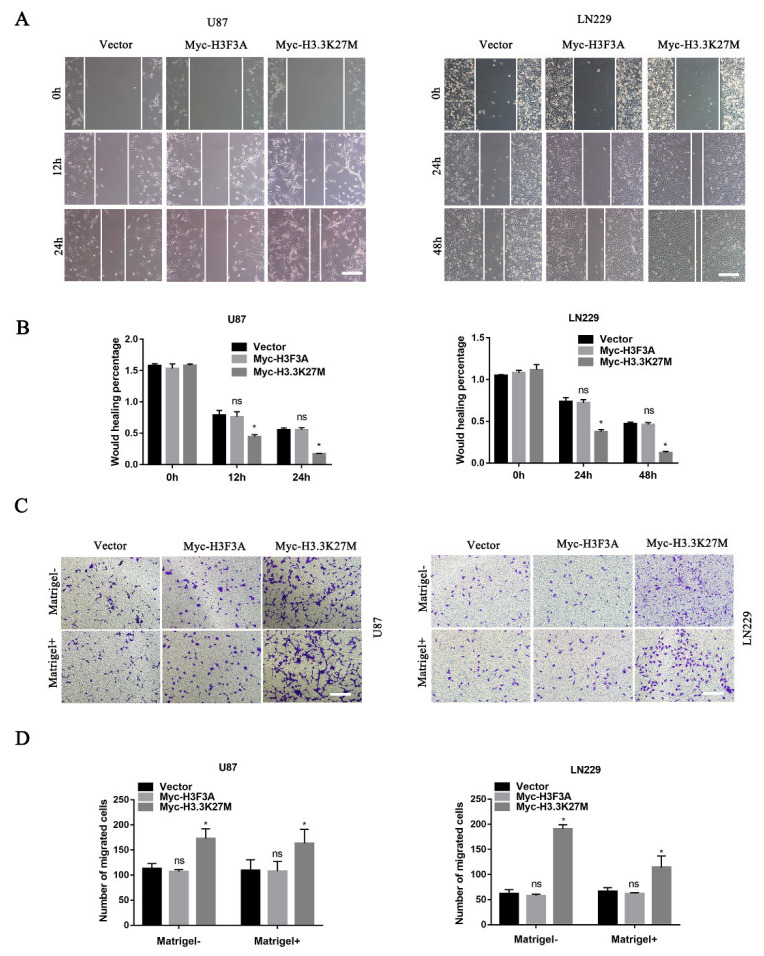
Wound healing and transwell assays showed that H3.3K27M overexpression promoted the invasion and migration of glioma *in vitro*. Representative images (**A**) and quantification (**B**) of wound healing results for U87 and LN229 cells. Representative images (**C**) and statistical graph (**D**) of the results of the transwell migration and invasion assays using U87 and LN229 cells. * *p* < 0.05; ns indicates no statistical significance.

Based on the aforementioned results, we hypothesized that H3.3K27M mainly affects glioma cell motility. Thus, we explored its roles in glioma cell migration and invasion. The wound healing assay showed that the migration ability of U87 and LN229 cells stably expressing H3.3K27M was markedly enhanced compared with that of the control or WT group (Figure 4A,B). The transwell migration assay (without Matrigel) produced similar results as the wound healing assay (Figure 4C). Additionally, the transwell invasion (with Matrigel) assay showed that the H3.3K27M group had a much stronger invasion ability than the control or WT group (Figure 4D).

### 3.4. H3.3K27M Positively Regulates the β-Catenin/USP1 Signaling Pathway

**Figure 5:**
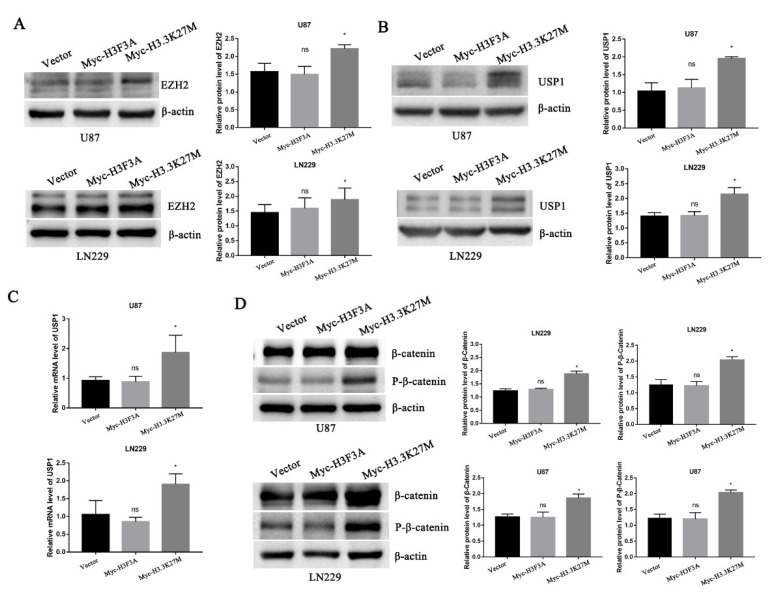
Western blotting and RT-PCR showed that H3.3K27M regulated the β-catenin/USP1/EZH2 signaling pathway. (**A**) Representative blots and statistical graph of the H3.3K27M-induced increase in EZH2 activity in U87 and LN229 cells. (**B**) Representative blots and histograms showing the quantitative analysis of the increase in USP1 protein levels induced by H3.3K27M in U87 and LN229 cells. (**C**) Quantification of USP1 mRNA levels in U87 and LN229 cells after stable H3.3K27M overexpression. (**D**) Representative blots and statistical graph of β-catenin and P-β-catenin (Ser675) levels in U87 and LN229 cells. * *p* < 0.05; ns indicates no statistical significance.

**Figure 6:**
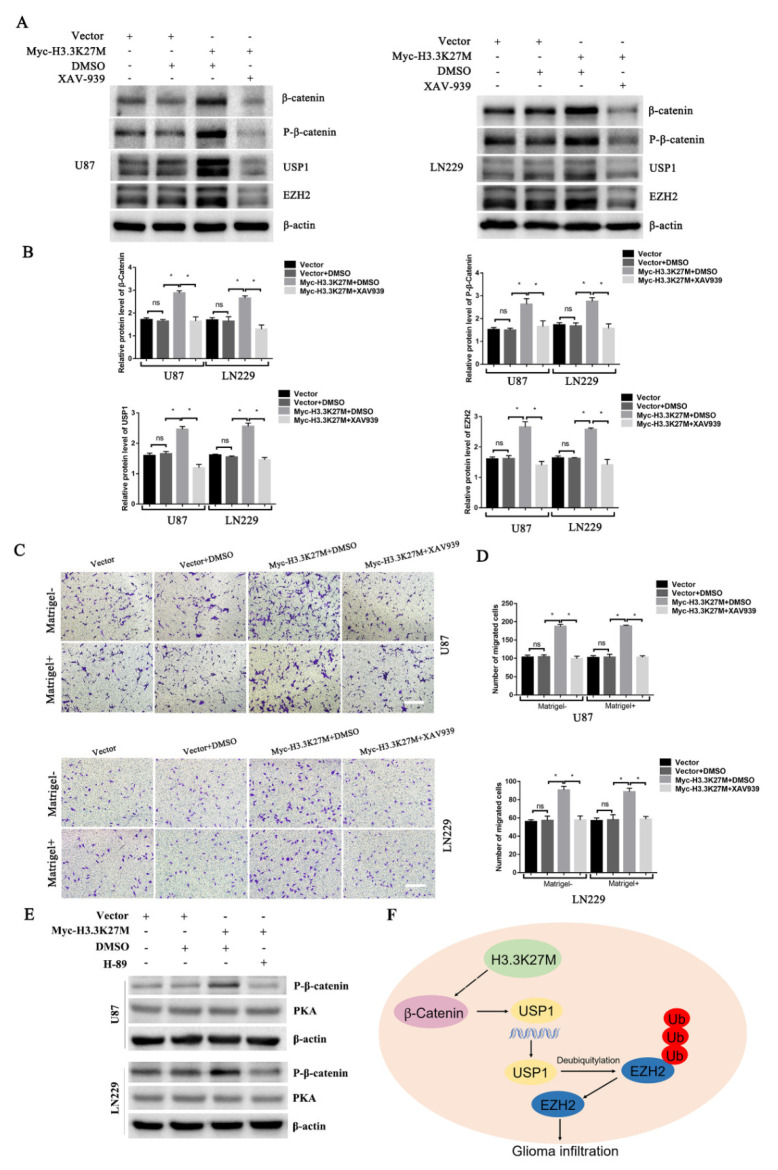
Western blotting and transwell assays showed that XAV-939 blocked the H3.3K27M-induced upregulation of β-catenin/USP1/EZH2 signaling and promotion of migration and invasion. Representative blots (**A**) and histograms (**B**) of β-catenin, P-β-catenin, USP1 and EZH2 levels after XAV-939 addition in U87 and LN229 cells. Representative images (**C**) and statistical graph (**D**) of transwell migration and invasion results after XAV-939 treatment of U87 and LN229 cells. The vehicle DMSO had no obvious effect on the signaling pathway. The concentration of XAV-939 was 20 μM for 48 h. (**E**) Western blotting assays showed that the PKA inhibitor H-89 blocks the H3.3K27M-induced increase in β-catenin (Ser675) phosphorylation. (**F**) Schematic model illustrating the role of H3.3K27M in glioma tumor development. * *p* < 0.05; ns indicates no statistical significance.

In 2019, researchers reported that EZH2 participates in regulating the TGF-β pathway via a novel pathway axis that is potentially relevant in regulating the metastasis and aggressiveness of GBM [18]. Recent studies have also shown that EZH2 is a potential therapeutic target for H3K27M-mutant pediatric gliomas [17]. We thus hypothesized that EZH2 expression is involved in H3K27M mutation-induced glioma cell migration and invasion. We therefore detected the expression of EZH2 protein by Western blotting. As expected, the EZH2 protein was significantly upregulated upon overexpression of the H3.3K27M mutant (Figure 5A). Recent studies have suggested that the EZH2 protein is mainly regulated by the ubiquitin-proteasome-dependent degradation pathway, which is directly stabilized by ubiquitin-specific protease 1 (USP1). Additionally, β-catenin is the main transcription factor that promotes the transcription of USP1 [19]. Based on this knowledge, we assessed whether EZH2 upregulation is attributed to the ꞵ-catenin-USP1 axis. We subsequently detected the USP1 protein and mRNA levels of USP1. Western blot and qRT-PCR results showed that the overexpression of H3.3K27M upregulated USP1 levels at both the mRNA and protein levels in U87 and LN229 cells (Figure 5B,C). Furthermore, the USP1 upstream transcription factor β-catenin was found to be more significantly activated, which was reflected in the significant increase in the levels of both total ꞵ-catenin and phosphorylated β-catenin upon H3.3K27M overexpression (Figure 5D). More importantly, the β-catenin inhibitor XAV-939 was used to perform rescue experiment and confirm that the H3.3K27M induced EZH2 upregulation depended on the β-catenin/USP1 pathway. Overexpression of H3.3K27M in U87 and LN229 cells significantly increased the levels of USP1 and EZH2, changes that were significantly blocked by the β-catenin inhibitor XAV-939 (Figure 6A,B). Functionally, the invasion and migration induced by the H3.3K27M mutant were also inhibited by XAV-939 (Figure 6C,D). In addition, the PKA inhibitor H-89 effectively blocked the H3.3K27M-induced increase in β-catenin (Ser675) phosphorylation (Figure 6E). Taken together, these results indicate that H3.3K27M plays an important role in promoting glioma cell migration and invasion by activating the ꞵ-catenin/USP1/EZH2 signaling pathway (Figure 6F).

## 4. Discussion

In our study, we assessed the H3.3K27M expression of in 28 human tissues. We found that three patients with high-grade glioma carried the H3.3K27M mutation. We performed DNA sequencing to confirm the existence of H3.3K27M in these three samples and found that the AAG codon at position 27 was mutated to the ATG codon. Then, we constructed stable cell lines expressing H3.3K27M using the H3F3A wild-type glioma cell lines U87 and LN229. In the animal experiment, we found that H3.3K27M had no obvious effect on the growth of glioma but promoted the infiltration of glioma cells. In parallel, H3.3K27M had no effect on classic kinase-related proliferation pathways, including the AKT, MEK1/2 and STAT3 pathways. As the glioblastoma microenvironment is multifaceted and is composed of soluble factors, extracellular matrix components and tissue-resident cell types along with resident or recruited immune cells, these components may also play a role [20]. This interesting phenomenon motivated us to investigate the role of H3.3K27M in the migration and invasion of cell lines in vitro. Wound healing and transwell assays revealed that H3.3K27M increased the migration and invasion of U87 and LN229 cells. Our study showed the mechanism by which H3.3K27M promotes migration and invasion. H3.3K27M significantly activated the β-catenin/USP1/EZH2 pathway in U87 and LN229 cells. Our study reveals the molecular mechanism by which the H3.3K27M mutation promotes the malignancy of mutated glioma and provides some direction for research on targeted drugs. The number of H3.3K27M-mutant glioma cases has also increased gradually in recent years [21,22], and patients with H3.3K27M-mutant glioma may benefit from those studies.

One of the three patients harboring this mutation was a 19-year-old female. We initially classified her as an adult; however, some pediatric cancers may not develop and be diagnosed until late in young adulthood. Therefore, the H3.3K27M frequency will decrease if we exclude this patient. This patient deserves further research. In addition, H3.3K27M mutant tumors almost exclusively occur in midline locations across the entire age spectrum [23]. In our study, the anatomical location of tumors in three patients was consistent with the literature, and the tumors were located in the fourth ventricle, thalamus or brainstem. On the other hand, among H3.3K27M mutants, the brainstem was the most common location in pediatric patients and the thalamus in adults [24]. Thus, the most common location of H3.3K27M tumors may differ between children and adults, but they were both mainly observed in midline locations. The role of H3.3K27M in young adults and older adults deserves more investigation in the future.

In 2015, researchers reported that although H3.3K27M mutations are frequently observed in brainstem and thalamic gliomas, these mutations often lead to a poor prognosis for patients with brainstem gliomas [25]. However, Karremann et al. showed that an anatomical midline location, histopathological grade and tumor resection range had no effect on the survival of H3.3K27M-mutated glioma [14]. Although the effect on the anatomical location on patient outcomes remains controversial, the poor prognosis and the lack of effective treatment for patients with H3.3K27M-mutated glioma are unmet clinical needs. Hence, the specific molecular mechanisms underlying H3.3 mutation-driven glioma have not been elucidated and should be further studied. The H3.3K27M mutation exists in glioma in children and adults and may lead to a poor prognosis. Thus, we wanted to investigate whether H3.3K27M affects the malignant behaviors of glioma. Since migration and invasion are classical malignant behaviors in the progression of tumor [26], we performed wound healing and transwell assays and found that the expression of H3.3K27M expression was related to the migration and invasion of glioma and may explain the poor prognosis of H3.3K27M glioma. The identification of an aberrantly activated pathway may provide a new therapeutic strategy for the precise targeting of H3.3K27M mutant gliomas.

EZH2 is the methyltransferase component of PRC2. H3.3K27M histones bind to the SET domain of EZH2, causing a global reduction in H3K27 dimethylation and trimethylation (H3K27me2/3) [27]. However, researchers reported that the H3.3K27M mutants showed increased H3K27 methylation at unique loci in SF7761 cells due to the recruitment of EZH2. Their genome-wide sequencing data also suggest that the disruption of the H3K27me3 profile by the H3.3K27M mutation may drive tumor formation in pediatric patients with diffuse intrinsic pontine glioma (DIPG) [28]. Li et al. elucidated a SOX4-dependent epithelial–mesenchymal transition (EMT)-inducing mechanism underlying MTA1-driven cancer metastasis and suggested a widespread TGF-β-MTA1-SOX4-EZH2 signaling axis that drives the EMT in various cancers [29]. Chen et al. identified a novel metastasis-promoting lncRNA, MRPL23-AS1, mediating the transcriptional silencing of E-cadherin by forming an RNA-protein complex with EZH2 [30]. EZH2 is also closely associated with the EMT of other tumors, such as pancreatic cancer, head and neck squamous cell carcinoma and esophageal cancer [31,32,33]. The EZH2 inhibitor MC4040 reverses the EMT and impairs cell migration and invasion, attenuating the glioma malignant phenotype [34]. Studies have also reported that EZH2 is involved in a novel miR-490-3p/TGIF2/TGFBR1 axis inducing migration and EMT in glioblastomas [18]. EZH2 is also associated with glioma proliferation and metastasis and has been regarded as a potential predictor and therapeutic target in glioma [35,36]. In general, EZH2 might be a marker associated with the EMT in glioma. Our study revealed that EZH2 is closely related to the migration and invasion of glioma and supports the hypothesis that EZH2 plays an important role in the EMT of glioma.

Researchers have focused on studying the underlying mechanisms of H3.3K27M glioma due to the poor prognosis of H3.3K27M-mutant glioma in children, and they have made some advances in clarifying the molecular mechanisms of DIPG. Mechanistically, studies have revealed that the JNK pathway, NOTCH pathway, RAS/MYC axis and Rb/E2F1 pathway participate in the development of H3.3K27M-positive gliomas [37,38,39,40]. As previously mentioned, EZH2 participates in the migration and invasion of glioma. Thus, we detected the expression of EZH2 after overexpressing H3.3K27M in U87 and LN229 cells. As a result, EZH2 expression increased with H3.3K27M overexpression. Abnormal ubiquitination and degradation are the main reasons for EZH2 protein accumulation, which is primarily regulated by the deubiquitinase USP1 [19]. We therefore focused on exploring the expression of USP1 and components of its upstream pathway. Our results showed that both the protein and mRNA levels of USP1 increased upon H3.3K27M overexpression.β-Catenin acts as both a transcriptional coregulator and an adaptor protein for intracellular adhesion [41]. Mechanistically, β-catenin/TCF4 activates the transcription of the deubiquitinase USP1, which then directly interacts with and deubiquitinates EZH2 [19]. USP1-mediated stabilization of EZH2 promotes the occurrence and development of glioma [19]. Furthermore, we demonstrated that the upregulation of USP1 was attributed to the activation of β-catenin, a classic signal transduction protein related to the malignant progression of glioma, in our study. Based on these results, H3.3K27M promotes glioma cell invasion and migration through the β-catenin/USP1 signaling pathway. Nevertheless, β-catenin/USP1/EZH2 has no obvious effect on the proliferation of glioma cells. As reported, the age of the patient and the specific location of the H3K27M tumor lead to different biological behaviors [42]. H3.3K27M-mediated gliomagenesis depends on PDGF signaling in a genetic mouse model [43]. Thus, we speculate that H3.3K27M has no direct effect on the proliferation of glioma, which might be due to the lack of special genetic mutations. In addition, the constructed cell lines rely on a different set of other mutations, which may influence the function of H3.3K27M. However, further experimental studies must be conducted to assess the role of H3.3K27M in the β-catenin pathway.

β-Catenin is phosphorylated at Ser675 by protein kinase A (PKA) [44,45]. Phosphorylation at Ser675 by PKA induces the nuclear accumulation of β-catenin and enhances its transcriptional activity (TCF/LEF transactivation) [44]. In our study, the level of phosphorylated β-catenin (Ser675) was significantly increased upon H3.3K27M overexpression (Figure 5D). Moreover, the PKA inhibitor H-89 inhibited the increased level of β-catenin phosphorylation caused by the H3.3K27M mutant (Figure 6E). Based on these results, we infer that Myc-H3.3K27M may be involved in β-catenin phosphorylation by regulating PKA activity. On the other hand, GSK-3β phosphorylates β-catenin at Ser33, Ser37 and Thr41 in the Wnt-signaling pathway [46]. This phosphorylation usually targets β-catenin for ubiquitination and degradation by the proteasome system [45]. In the canonical Wnt signaling pathway, only phosphorylated β-catenin could be degraded by ubiquitination. However, new research findings reveal that nonphosphorylated β-catenin is directly ubiquitinated as well, which is mediated by another E3 called Siah-1 (Seven in absentia homolog 1) [47]. In our study, total β-catenin was also found to be upregulated upon H3.3K27M overexpression (Figure 5D). This result seems to be due to reduced ubiquitination and degradation. Thus, we speculate that Myc-H3.3K27M may participate in the ubiquitination and degradation process regulated by Siah-1. 

## 5. Conclusions

In conclusion, our research confirmed that H3.3K27M is linked to the β-catenin/USP1 pathway. This signaling pathway is also involved in the malignant activity of human glioma. Our research results may provide some basis for studying the function of H3.3K27M in adult glioma. The β-catenin/USP1 pathway may be a potential target in H3.3K27M mutant glioma. This study may provide new insights for studies of drug targeting and biomarkers of gliomas with the H3.3K27M mutation. However, we should conduct more exhaustive studies to explore the detailed mechanisms of H3.3K27M in future projects, such as whether H3.3K27M directly binds to β-catenin to activate this pathway.

## Data Availability

All datasets supporting the conclusions contained in the present report are included in the manuscript.

## Supplementary Materials

The following supporting information can be downloaded at: https://www.mdpi.com/article/10.3390/cancers14194836/s1. Table S1: Patient Demographics.

## References

[1] Wesseling P., Capper D. (2017). WHO 2016 Classification of gliomas. Neuropathol. Appl. Neurobiol..

[2] Ostrom Q.T., Gittleman H., Stetson L., Virk S.M., Barnholtz-Sloan J.S. (2014). Epidemiology of Gliomas. Current Understanding and Treatment of Gliomas.

[3] Bush N.A.O., Chang S.M., Berger M.S. (2016). Current and future strategies for treatment of glioma. Neurosurg. Rev..

[4] Montemurro N., Fanelli G.N., Scatena C., Ortenzi V., Pasqualetti F., Mazzanti C.M., Morganti R., Paiar F., Naccarato A.G., Perrini P. (2021). Surgical outcome and molecular pattern characterization of recurrent glioblastoma multiforme: A single-center retrospective series. Clin. Neurol. Neurosurg..

[5] Ma C., Nguyen H.P.T., Jones J.J., Stylli S.S., Whitehead C.A., Paradiso L., Luwor R.B., Areeb Z., Hanssen E., Cho E. (2022). Extracellular Vesicles Secreted by Glioma Stem Cells Are Involved in Radiation Resistance and Glioma Progression. Int. J. Mol. Sci..

[6] Audia J.E., Campbell R.M. (2016). Histone Modifications and Cancer. Cold Spring Harb. Perspect. Biol..

[7] Kouzarides T. (2007). Chromatin modifications and their function. Cell.

[8] Bannister A.J., Kouzarides T. (2011). Regulation of chromatin by histone modifications. Cell Res..

[9] Xiang Y., Yan K., Zheng Q., Ke H., Cheng J., Xiong W., Shi X., Wei L., Zhao M., Yang F. (2019). Histone Demethylase KDM4B Promotes DNA Damage by Activating Long Interspersed Nuclear Element-1. Cancer Res..

[10] Shi L., Wen H., Shi X. (2016). The Histone Variant H3.3 in Transcriptional Regulation and Human Disease. J. Mol. Biol..

[11] Schwartzentruber J., Korshunov A., Liu X.-Y., Jones D.T.W., Pfaff E., Jacob K., Sturm D., Fontebasso A.M., Khuong-Quang D.-A., Tönjes M. (2012). Driver mutations in histone H3.3 and chromatin remodelling genes in paediatric glioblastoma. Nature.

[12] Gessi M., Capper D., Sahm F., Huang K., von Deimling A., Tippelt S., Fleischhack G., Scherbaum D., Alfer J., Juhnke B.-O. (2016). Evidence of H3 K27M mutations in posterior fossa ependymomas. Acta Neuropathol..

[13] Joyon N., Tauziède-Espariat A., Alentorn A., Giry M., Castel D., Capelle L., Zanello M., Varlet P., Bielle F. (2017). K27M mutation in*H3F3A*in ganglioglioma grade I with spontaneous malignant transformation extends the histopathological spectrum of the histone H3 oncogenic pathway. Neuropathol. Appl. Neurobiol..

[14] Karremann M., Gielen G.H., Hoffmann M., Wiese M., Colditz N., Warmuth-Metz M., Bison B., Claviez A., Van Vuurden D.G., Von Bueren A. (2017). Diffuse high-grade gliomas with H3 K27M mutations carry a dismal prognosis independent of tumor location. Neuro-Oncology.

[15] Margueron R., Reinberg D. (2011). The Polycomb complex PRC2 and its mark in life. Nature.

[16] Laugesen A., Højfeldt J.W., Helin K. (2019). Molecular Mechanisms Directing PRC2 Recruitment and H3K27 Methylation. Mol. Cell.

[17] Mohammad F., Weissmann S., Leblanc B., Pandey D.P., Hojfeldt J., Comet I., Zheng C., Johansen J.V., Rapin N., Porse N.R.B.T. (2017). EZH2 is a potential therapeutic target for H3K27M-mutant pediatric gliomas. Nat. Med..

[18] Vinchure O.S., Sharma V., Tabasum S., Ghosh S., Singh R.P., Sarkar C., Kulshreshtha R. (2019). Polycomb complex mediated epigenetic reprogramming alters TGF-β signaling via a novel EZH2/miR-490/TGIF2 axis thereby inducing migration and EMT potential in glioblastomas. Int. J. Cancer.

[19] Ma L., Lin K., Chang G., Chen Y., Yue C., Guo Q., Zhang S., Jia Z., Huang T.T., Zhou A. (2019). Aberrant Activation of β-Catenin Signaling Drives Glioma Tumorigenesis via USP1-Mediated Stabilization of EZH2. Cancer Res..

[20] Fanelli G., Grassini D., Ortenzi V., Pasqualetti F., Montemurro N., Perrini P., Naccarato A., Scatena C. (2021). Decipher the Glioblastoma Microenvironment: The First Milestone for New Groundbreaking Therapeutic Strategies. Genes.

[21] Maimaiti B., Mijiti S., Jiang T., Xie Y., Zhao W., Cheng Y., Meng H. (2022). Case Report: H3K27M-Mutant Glioblastoma Simultaneously Present in the Brain and Long-Segment Spinal Cord Accompanied by Acute Pulmonary Embolism. Front. Oncol..

[22] Niu X., Wang C., Zhou X., Yang Y., Liu Y., Zhang Y., Mao Q. (2020). Pineal Region Glioblastomas: Clinical Characteristics, Treatment, and Survival Outcome. World Neurosurg..

[23] Sturm D., Witt H., Hovestadt V., Khuong-Quang D.-A., Jones D.T., Konermann C., Pfaff E., Tönjes M., Sill M., Bender S. (2012). Hotspot Mutations in H3F3A and IDH1 Define Distinct Epigenetic and Biological Subgroups of Glioblastoma. Cancer Cell.

[24] Manjunath N., Jha P., Singh J., Raheja A., Kaur K., Suri A., Garg A., Sharma M.C., Sarkar C., Mohan M. (2020). Clinico-pathological and molecular characterization of diffuse midline gliomas: Is there a prognostic significance?. Neurol. Sci..

[25] Feng J., Hao S., Pan C., Wang Y., Wu Z., Zhang J., Yan H., Zhang L., Wan H. (2015). The H3.3 K27M mutation results in a poorer prognosis in brainstem gliomas than thalamic gliomas in adults. Hum. Pathol..

[26] Yeo M.S., Subhash V.V., Suda K., Balcıoğlu H.E., Zhou S., Thuya W.L., Loh X.Y., Jammula S., Peethala P.C., Tan S.H. (2019). FBXW5 Promotes Tumorigenesis and Metastasis in Gastric Cancer via Activation of the FAK-Src Signaling Pathway. Cancers.

[27] Lewis P.W., Müller M.M., Koletsky M.S., Cordero F., Lin S., Banaszynski L.A., Garcia B.A., Muir T.W., Becher O.J., Allis C.D. (2013). Inhibition of PRC2 Activity by a Gain-of-Function H3 Mutation Found in Pediatric Glioblastoma. Science.

[28] Chan K.-M., Fang D., Gan H., Hashizume R., Yu C., Schroeder M., Gupta N., Mueller S., James C.D., Jenkins R. (2013). The histone H3.3K27M mutation in pediatric glioma reprograms H3K27 methylation and gene expression. Genes Dev..

[29] Li L., Liu J., Xue H., Li C., Liu Q., Zhou Y., Wang T., Wang H., Qian H., Wen T. (2019). A TGF-β-MTA1-SOX4-EZH2 signaling axis drives epithelial–mesenchymal transition in tumor metastasis. Oncogene.

[30] Chen C.-W., Fu M., Du Z.-H., Zhao F., Yang W.-W., Xu L.-H., Li S.-L., Ge X.-Y. (2020). Long Noncoding RNA MRPL23-AS1 Promotes Adenoid Cystic Carcinoma Lung Metastasis. Cancer Res..

[31] Ma J., Zhang J., Weng Y.-C., Wang J.-C. (2018). EZH2-Mediated microRNA-139-5p Regulates Epithelial-Mesenchymal Transition and Lymph Node Metastasis of Pancreatic Cancer. Mol. Cells.

[32] Zhao M., Hu X., Xu Y., Wu C., Chen J., Ren Y., Kong L., Sun S., Zhang L., Jin R. (2019). Targeting of EZH2 inhibits epithelial-mesenchymal transition in head and neck squamous cell carcinoma via regulating the STAT3/VEGFR2 axis. Int. J. Oncol..

[33] Zhang S., Liao W., Wu Q., Huang X., Pan Z., Chen W., Gu S., Huang Z., Wang Y., Tang X. (2020). LINC00152 upregulates ZEB1 expression and enhances epithelial-mesenchymal transition and oxaliplatin resistance in esophageal cancer by interacting with EZH2. Cancer Cell Int..

[34] Stazi G., Taglieri L., Nicolai A., Romanelli A., Fioravanti R., Morrone S., Sabatino M., Ragno R., Taurone S., Nebbioso M. (2019). Dissecting the role of novel EZH2 inhibitors in primary glioblastoma cell cultures: Effects on proliferation, epithelial-mesenchymal transition, migration, and on the pro-inflammatory phenotype. Clin. Epigenetics.

[35] Zhang Y., Wang J., An W., Chen C., Wang W., Zhu C., Chen F., Chen H., Zheng W., Gong J. (2019). MiR-32 Inhibits Proliferation and Metastasis by Targeting EZH2 in Glioma. Technol. Cancer Res. Treat..

[36] Chen Y., Hou S., Jiang R., Sun J., Cheng C., Qian Z. (2020). EZH2 is a potential prognostic predictor of glioma. J. Cell. Mol. Med..

[37] Delaney K., Strobino M., Wenda J.M., Pankowski A., Steiner F.A. (2019). H3.3K27M-induced chromatin changes drive ectopic replication through misregulation of the JNK pathway in C. elegans. Nat. Commun..

[38] Chen K.-Y., Bush K., Klein R.H., Cervantes V., Lewis N., Naqvi A., Carcaboso A.M., Lechpammer M., Knoepfler P.S. (2020). Reciprocal H3.3 gene editing identifies K27M and G34R mechanisms in pediatric glioma including NOTCH signaling. Commun. Biol..

[39] Pajovic S., Siddaway R., Bridge T., Sheth J., Rakopoulos P., Kim B., Ryall S., Agnihotri S., Phillips L., Yu M. (2020). Epigenetic activation of a RAS/MYC axis in H3.3K27M-driven cancer. Nat. Commun..

[40] Ehteda A., Simon S., Franshaw L., Giorgi F.M., Liu J., Joshi S., Rouaen J.R., Pang C.N.I., Pandher R., Mayoh C. (2021). Dual targeting of the epigenome via FACT complex and histone deacetylase is a potent treatment strategy for DIPG. Cell Rep..

[41] Pan X., Ma L., Wang J. (2019). The clinicopathological significance and prognostic value of β-catenin Ser45-phosphorylation expression in esophageal squamous cell carcinoma. Int. J. Clin. Exp. Pathol..

[42] Daoud E.V., Rajaram V., Cai C., Oberle R.J., Martin G.R., Raisanen J.M., White C., Foong C., Mickey B., Pan E. (2018). Adult Brainstem Gliomas With H3K27M Mutation: Radiology, Pathology, and Prognosis. J. Neuropathol. Exp. Neurol..

[43] Cordero F.J., Huang Z., Grenier C., He X., Hu G., McLendon R.E., Murphy S.K., Hashizume R., Becher O.J. (2017). Histone H3.3K27M Represses *p16* to Accelerate Gliomagenesis in a Murine Model of DIPG. Mol. Cancer Res..

[44] Taurin S., Sandbo N., Qin Y., Browning D., Dulin N.O. (2006). Phosphorylation of β-Catenin by Cyclic AMP-dependent Protein Kinase. J. Biol. Chem..

[45] Hino S.-I., Tanji C., Nakayama K.I., Kikuchi A. (2005). Phosphorylation of β-Catenin by Cyclic AMP-Dependent Protein Kinase Stabilizes β-Catenin through Inhibition of Its Ubiquitination. Mol. Cell. Biol..

[46] Yost C., Torres M., Miller J.R., Huang E., Kimelman D., Moon R.T. (1996). The axis-inducing activity, stability, and subcellular distribution of beta-catenin is regulated in Xenopus embryos by glycogen synthase kinase 3. Genes Dev..

[47] Dimitrova Y., Li J., Lee Y.-T., Rios-Esteves J., Friedman D.B., Choi H.-J., Weis W., Wang C.-Y., Chazin W.J. (2010). Direct Ubiquitination of β-Catenin by Siah-1 and Regulation by the Exchange Factor TBL1. J. Biol. Chem..

