# Supplementary material for: H3F3A K27M Mutation Promotes the Infiltrative Growth of High-Grade Glioma in Adults by Activating β-Catenin/USP1 Signaling
Source: Cancers (Basel). 2022 Oct 3;14(19):4836. doi: 10.3390/cancers14194836 (PMC9563249; doi:10.3390/cancers14194836)
Supplement: Supplementary file 1 [file cancers-14-04836-s001.zip › cancers-1886589-original-images.pdf]

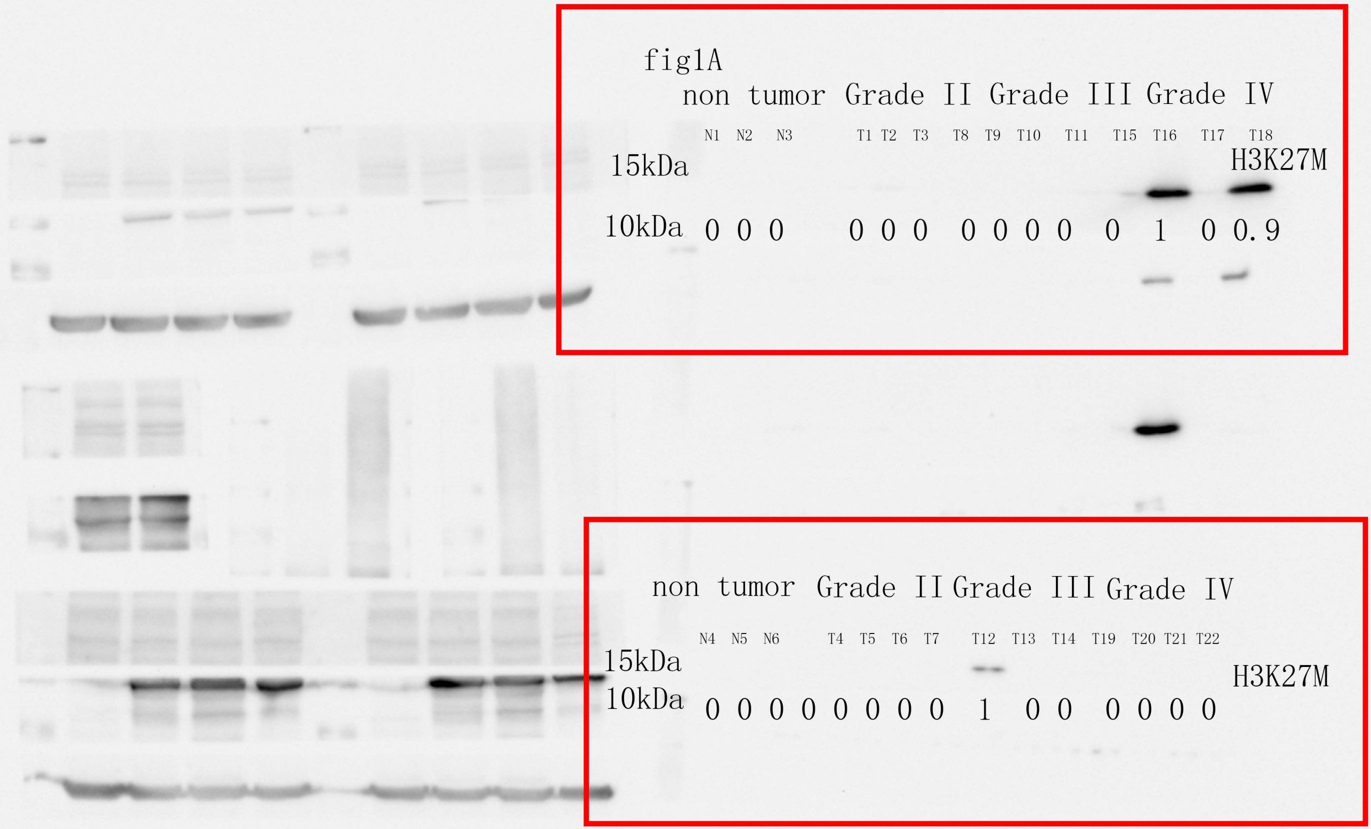
fig1A
non tumor Grade II Grade III Grade IV
N1 N2 N3 T1 T2 T3 T8 T9 T10 T11 T15 T16 T17 T18
15kDa
H3K27M
10kDa
0 0 0 0 0 0 0 0 0 0 0 1 0 0.9
non tumor Grade II Grade III Grade IV
N4 N5 N6 T4 T5 T6 T7 T12 T13 T14 T19 T20 T21 T22
15kDa
10kDa
H3K27M
0 0 0 0 0 0 0 0 1 0 0 0 0 0 0

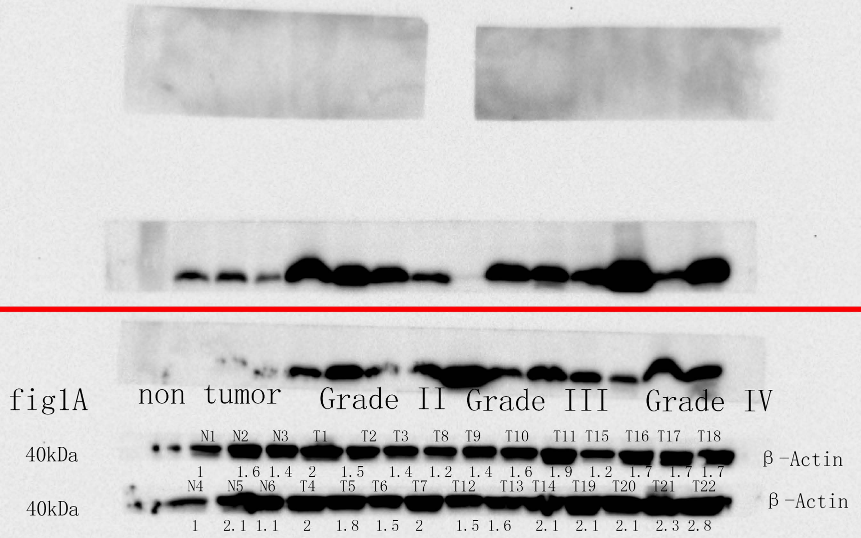
fig1A
non tumor
Grade II
Grade III
Grade IV
40kDa
N1 N2 N3 T1 T2 T3 T8 T9 T10 T11 T15 T16 T17 T18
β -Actin
1 1.6 1.4 2 1.5 1.4 1.2 1.4 1.6 1.9 1.2 1.7 1.7 1.7
40kDa
N4 N5 N6 T4 T5 T6 T7 T12 T13 T14 T19 T20 T21 T22
β -Actin
1 2.1 1.1 2 1.8 1.5 2 1.5 1.6 2.1 2.1 2.1 2.3 2.8

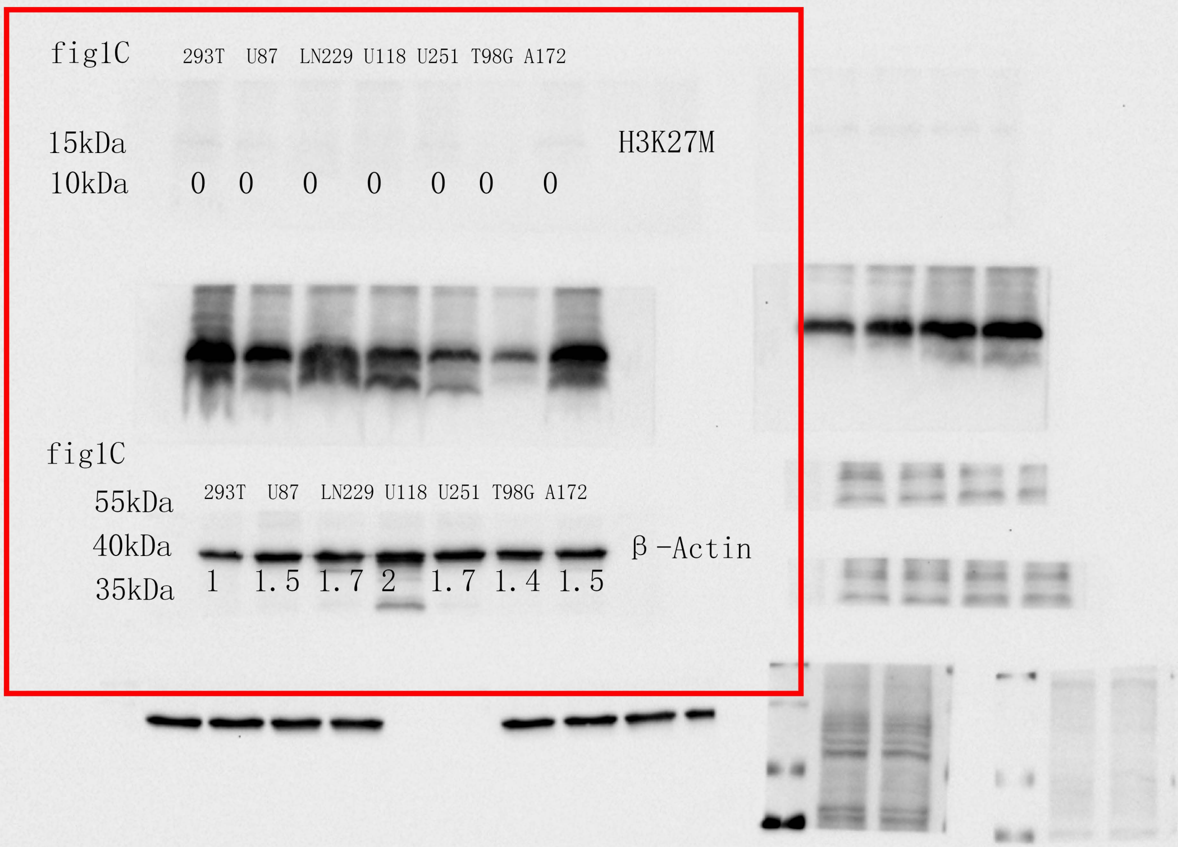
fig1C
293T U87 LN229 U118 U251 T98G A172
15kDa
10kDa
H3K27M
0 0 0 0 0 0 0
fig1C
55kDa
293T U87 LN229 U118 U251 T98G A172
40kDa
β-Actin
35kDa
1 1.5 1.7 2 1.7 1.4 1.5

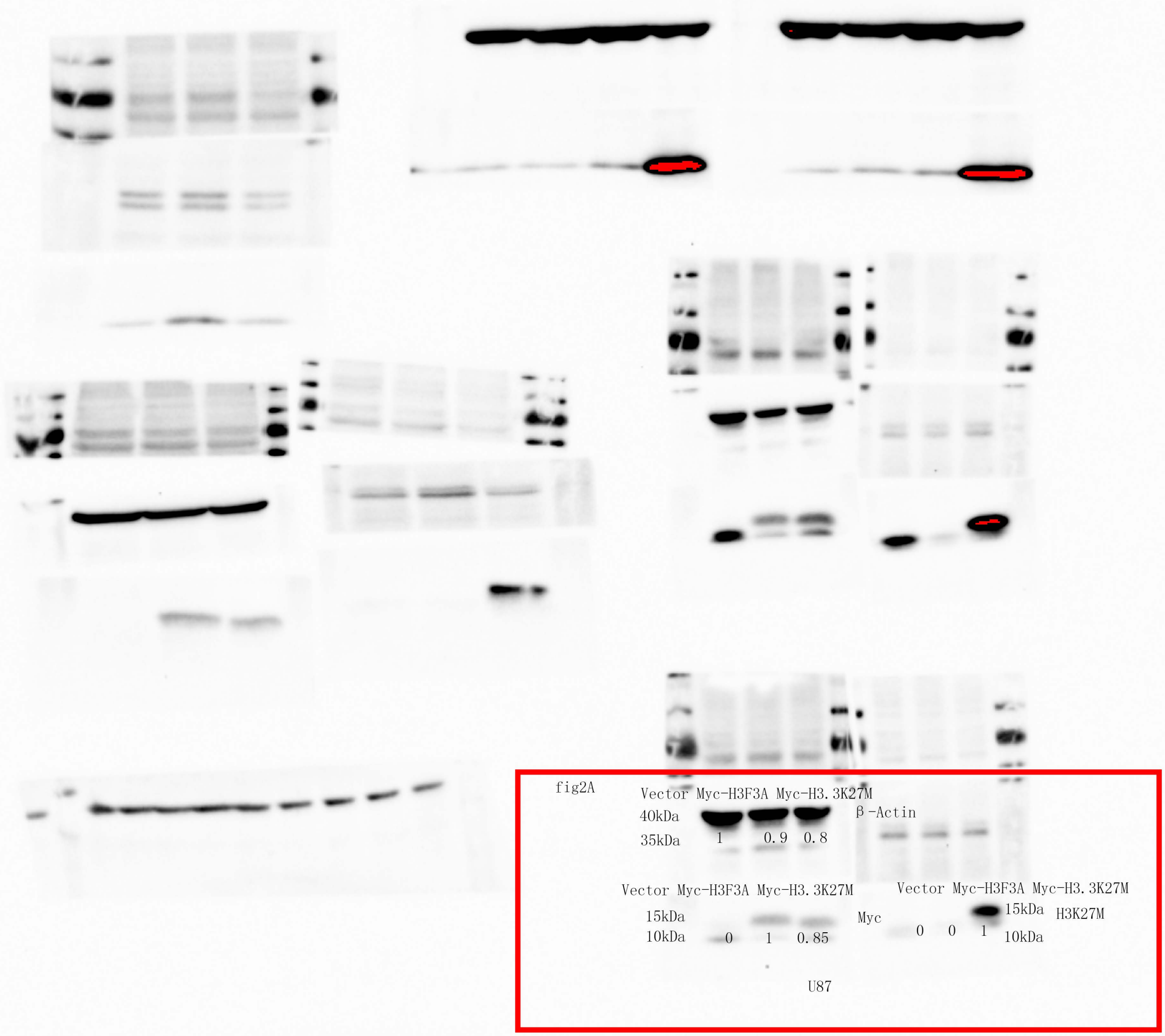
fig2A
Vector Myc-H3F3A Myc-H3.3K27M
40kDa
35kDa
1
0.9
0.8
β-Actin
Vector Myc-H3F3A Myc-H3.3K27M
15kDa
10kDa
0
1
0.85
Myc
Vector Myc-H3F3A Myc-H3.3K27M
0
0
1
15kDa
10kDa
H3K27M
U87

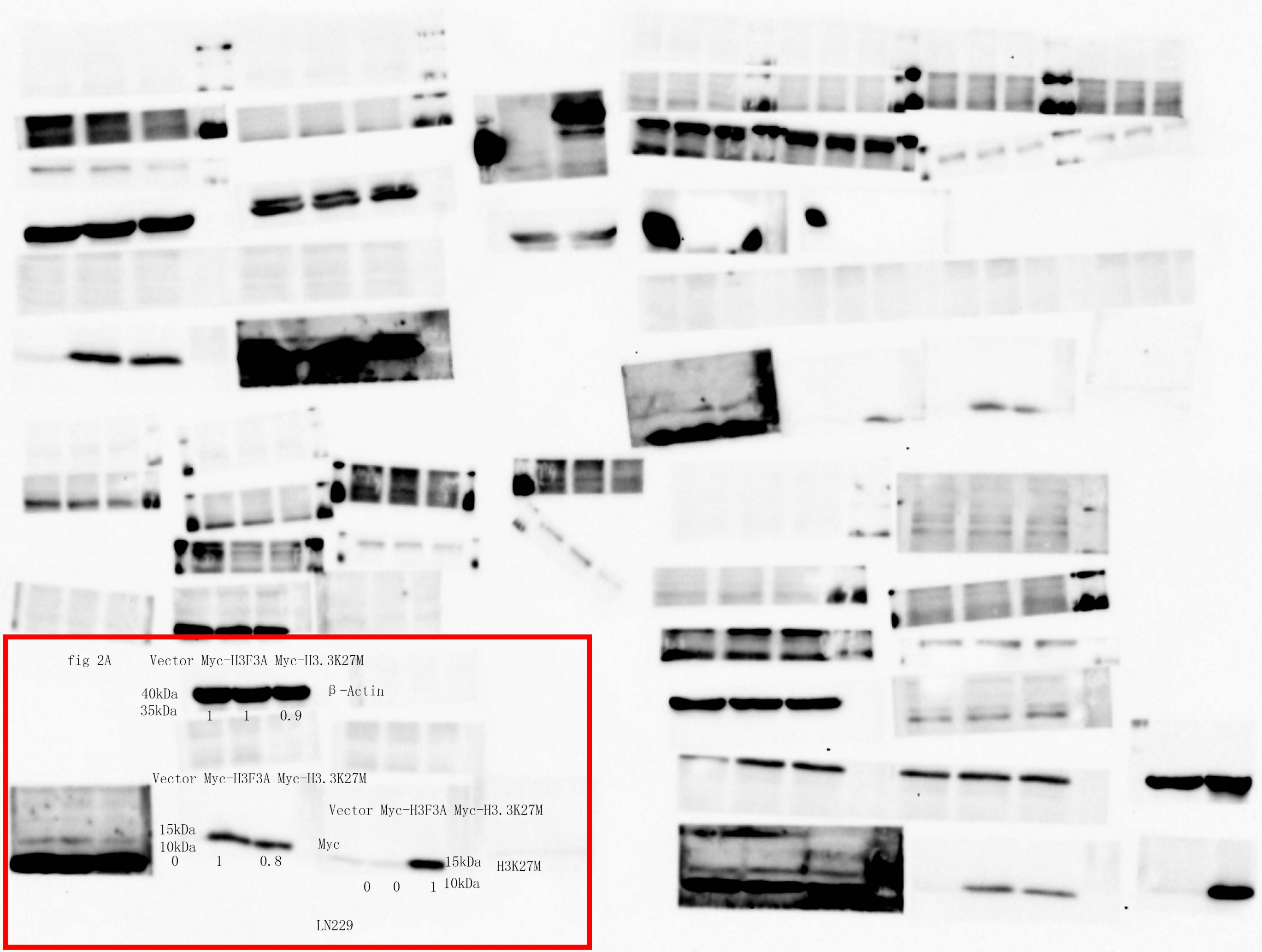
fig 2A
Vector Myc-H3F3A Myc-H3.3K27M
40kDa
35kDa
β-Actin
1 1 0.9
Vector Myc-H3F3A Myc-H3.3K27M
15kDa
10kDa
0 1 0.8
Myc
Vector Myc-H3F3A Myc-H3.3K27M
15kDa
10kDa
H3K27M
0 0 1
LN229

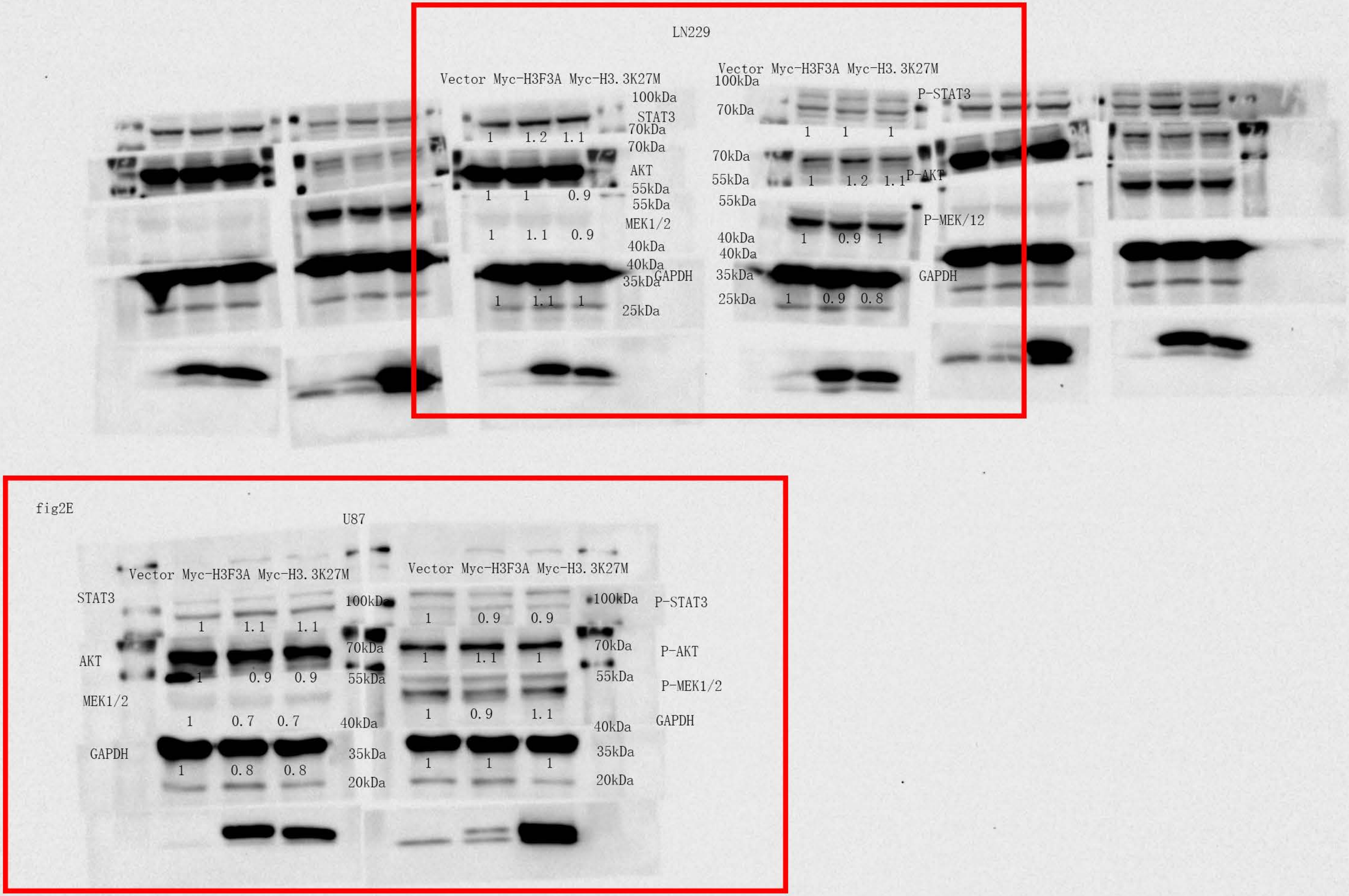

LN229
Vector Myc-H3F3A Myc-H3.3K27M
100kDa
STAT3
1 1.2 1.1
70kDa
70kDa
AKT
1 1 0.9
55kDa
55kDa
MEK1/2
1 1.1 0.9
40kDa
40kDa
35kDa
GAPDH
1 1.1 1
25kDa
Vector Myc-H3F3A Myc-H3.3K27M
100kDa
70kDa
P-STAT3
1 1 1
70kDa
55kDa
1 1.2 1.1
P-AKT
55kDa
P-MEK/12
40kDa
1 0.9 1
40kDa
35kDa
GAPDH
25kDa
1 0.9 0.8
fig2E
U87
Vector Myc-H3F3A Myc-H3.3K27M
STAT3
1 1.1 1.1
100kDa
70kDa
AKT
1 0.9 0.9
55kDa
MEK1/2
1 0.7 0.7
40kDa
GAPDH
35kDa
1 0.8 0.8
20kDa
Vector Myc-H3F3A Myc-H3.3K27M
100kDa
P-STAT3
1 0.9 0.9
70kDa
P-AKT
1 1.1 1
55kDa
P-MEK1/2
1 0.9 1.1
40kDa
GAPDH
35kDa
1 1 1
20kDa

fig5A U87

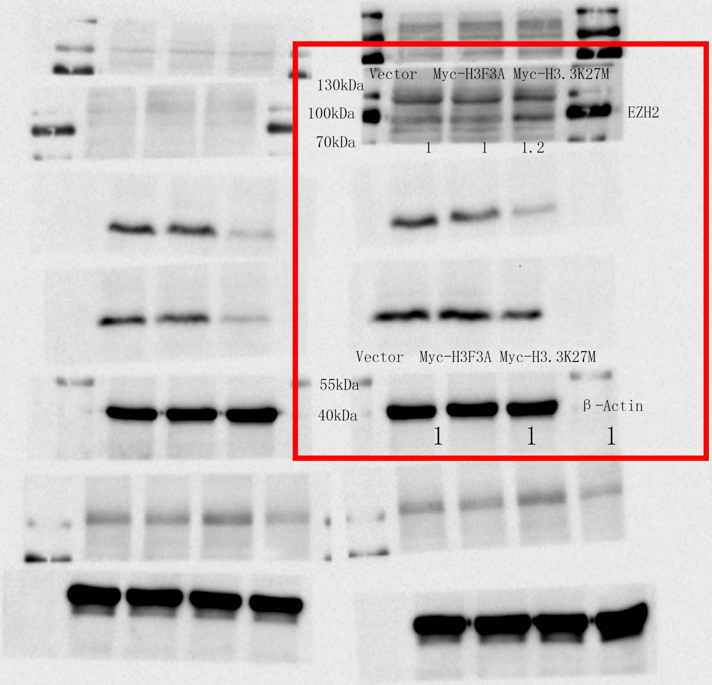
Vector Myc-H3F3A Myc-H3.3K27M
130kDa
100kDa
EZH2
70kDa
1 1 1.2
Vector Myc-H3F3A Myc-H3.3K27M
55kDa
β-Actin
40kDa
1 1 1

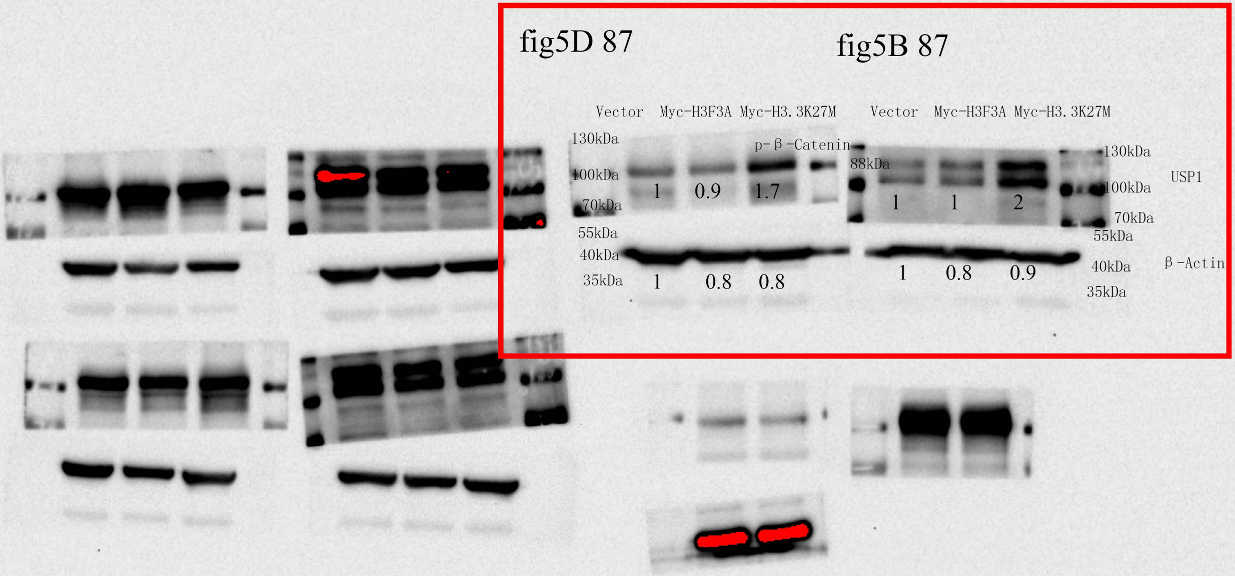
fig5D 87
fig5B 87
Vector
Myc-H3F3A
Myc-H3.3K27M
Vector
Myc-H3F3A
Myc-H3.3K27M
130kDa
100kDa
70kDa
55kDa
40kDa
35kDa
p-β-Catenin
1
0.9
1.7
1
0.8
0.8
88kDa
1
1
2
1
0.8
0.9
130kDa
100kDa
70kDa
55kDa
40kDa
35kDa
USP1
β-Actin

fig5A U87

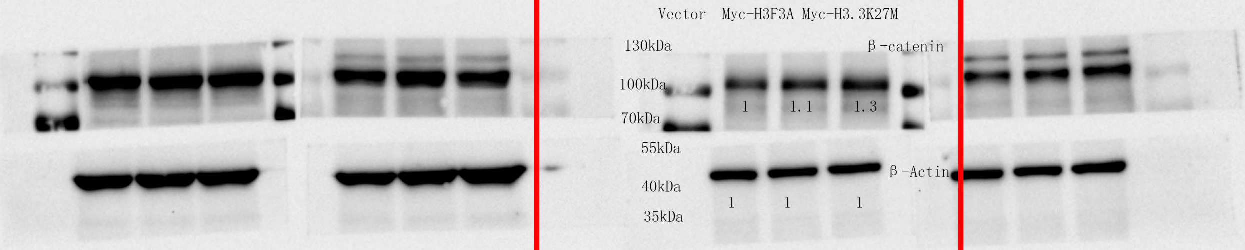
Vector
Myc-H3F3A
Myc-H3.3K27M
130kDa
100kDa
70kDa
55kDa
40kDa
35kDa
β-catenin
1
1.1
1.3
β-Actin
1
1
1

fig5A,D LN229

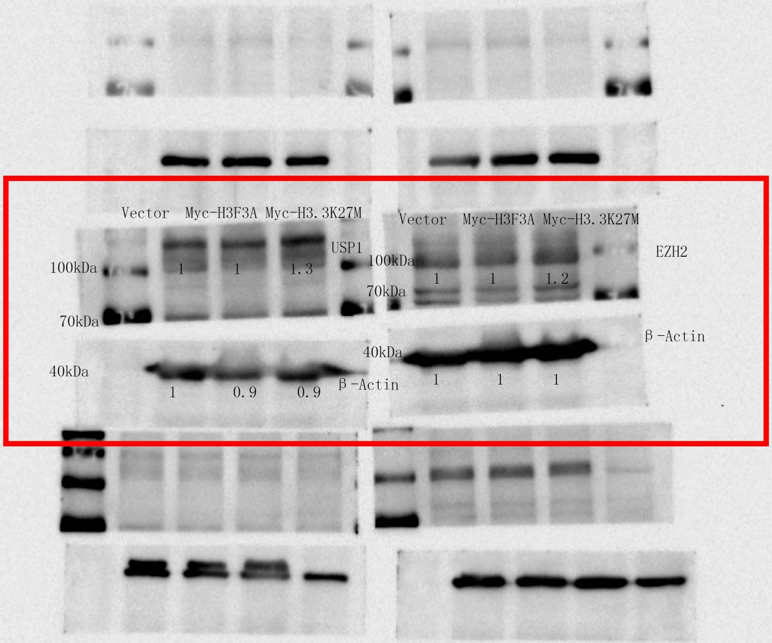

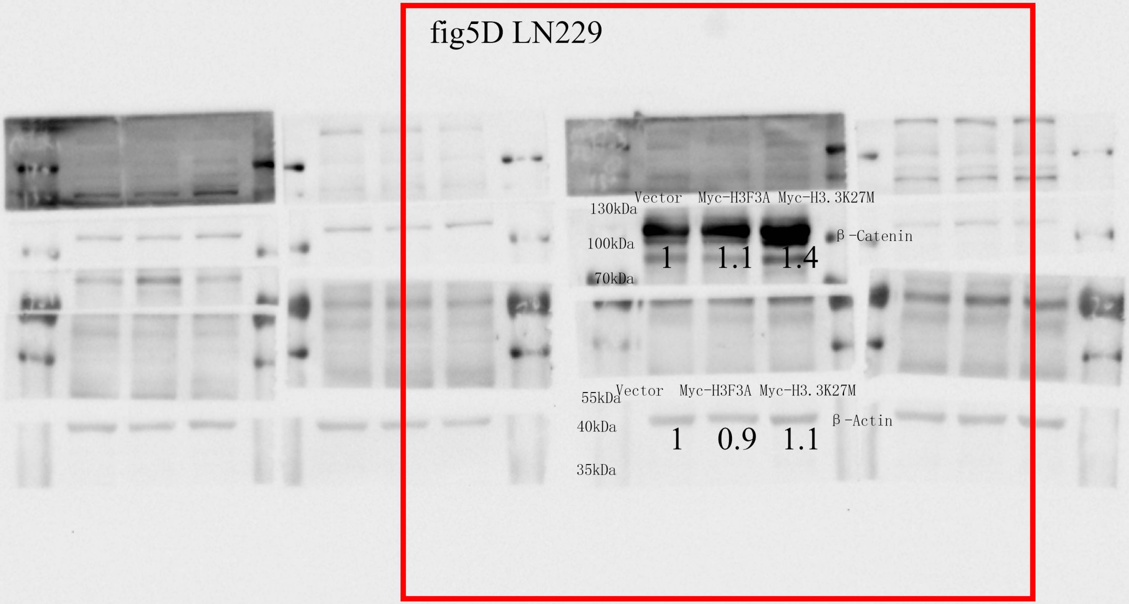
fig5D LN229
130kDa
Vector
Myc-H3F3A
Myc-H3.3K27M
100kDa
β-Catenin
1
1.1
1.4
70kDa
55kDa
Vector
Myc-H3F3A
Myc-H3.3K27M
40kDa
β-Actin
1
0.9
1.1
35kDa

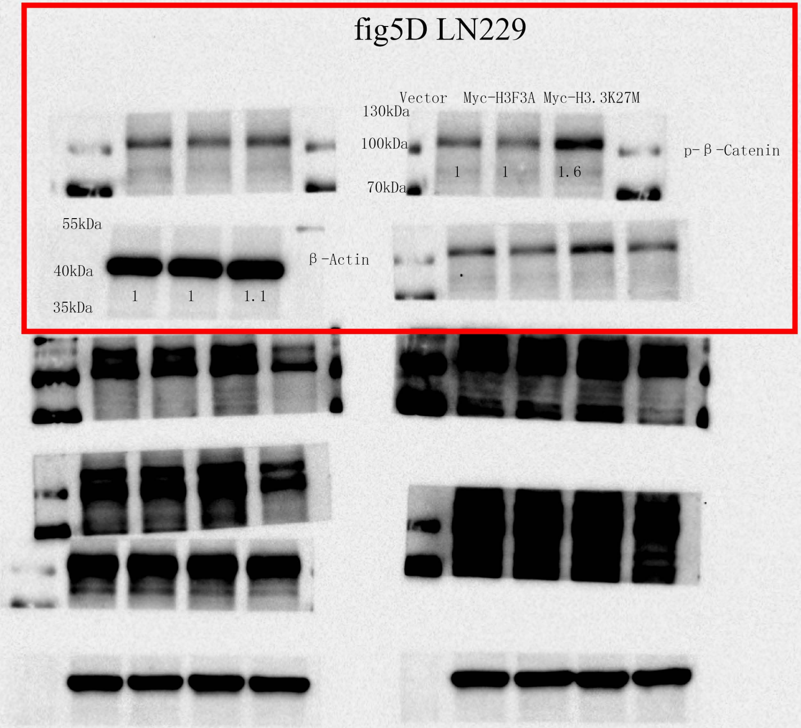
fig5D LN229
Vector
Myc-H3F3A
Myc-H3.3K27M
130kDa
100kDa
70kDa
p-β-Catenin
1
1
1.6
55kDa
40kDa
35kDa
β-Actin
1
1
1.1

fig5E U87

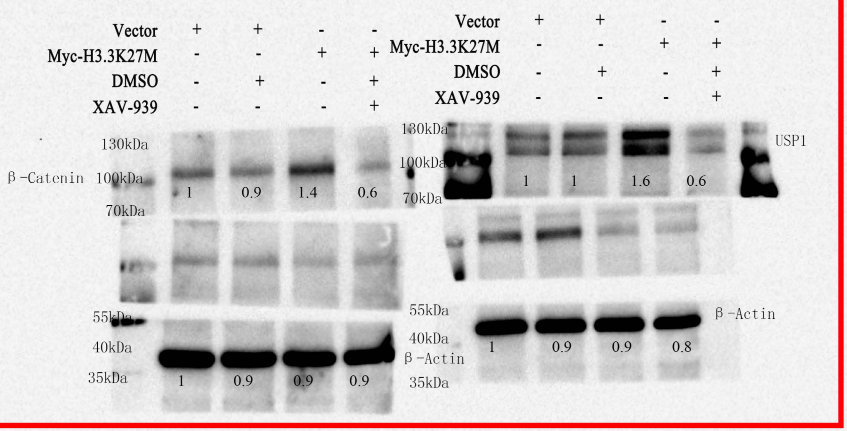
Vector + + - -
Myc-H3.3K27M - - + +
DMSO - + - +
XAV-939 - - - +
130kDa
β-Catenin 100kDa
70kDa
1 0.9 1.4 0.6
55kDa
40kDa
35kDa
1 0.9 0.9 0.9
Vector + + - -
Myc-H3.3K27M - - + +
DMSO - + - +
XAV-939 - - - +
130kDa
100kDa
70kDa
USP1
1 1 1.6 0.6
55kDa
40kDa
β-Actin
35kDa
β-Actin
1 0.9 0.9 0.8

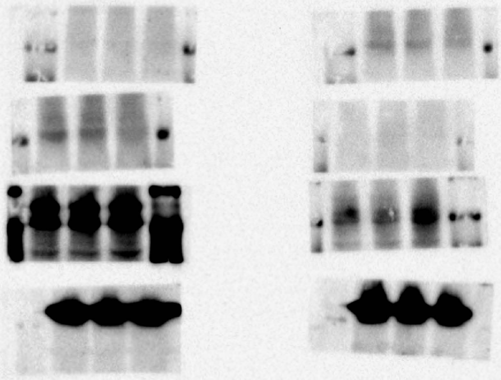

fig5E U87

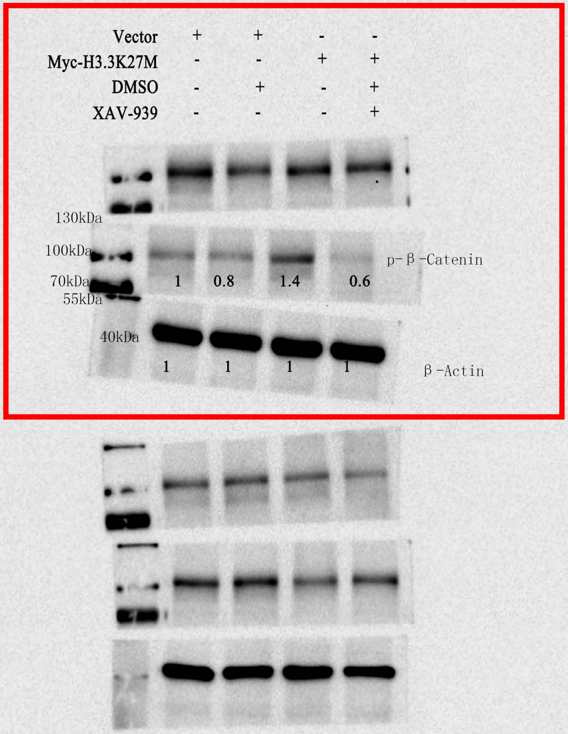
Vector + + - -
Myc-H3.3K27M - - + +
DMSO - + - +
XAV-939 - - - +
130kDa
100kDa
70kDa
55kDa
40kDa
p-β-Catenin
1 0.8 1.4 0.6
β-Actin
1 1 1 1

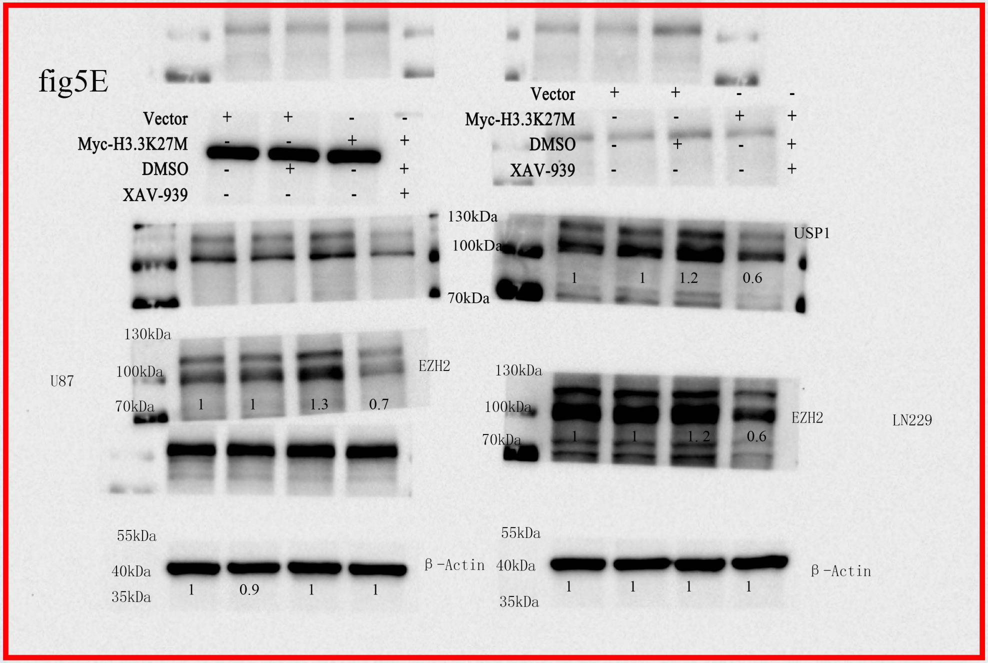
fig5E
Vector + + - -
Myc-H3.3K27M - - + +
DMSO - + - +
XAV-939 - - - +
130kDa
100kDa
70kDa
USP1
1 1 1.2 0.6
U87
EZH2
1 1 1.3 0.7
LN229
1 1 1.2 0.6
55kDa
40kDa
35kDa
β-Actin
1 0.9 1 1
1 1 1 1

fig5E LN229

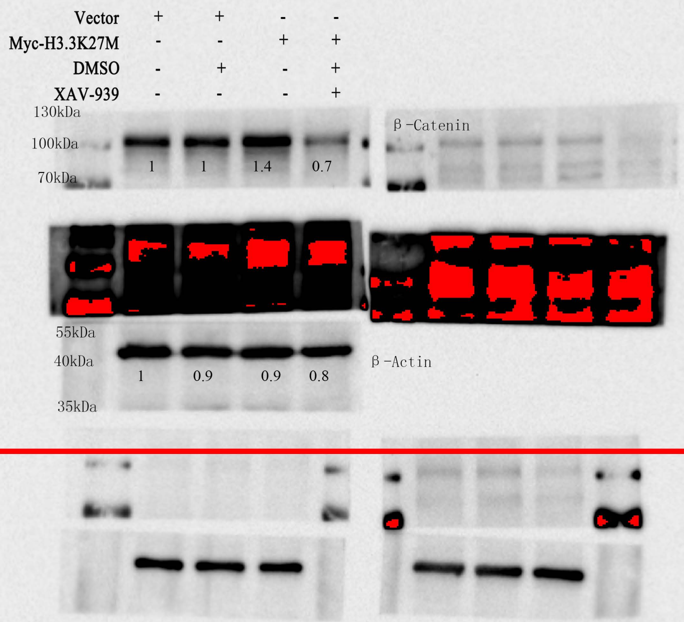
Vector + + - -
Myc-H3.3K27M - - + +
DMSO - + - +
XAV-939 - - - +
130kDa
100kDa
70kDa
1 1 1.4 0.7
β-Catenin
55kDa
40kDa
35kDa
1 0.9 0.9 0.8
β-Actin

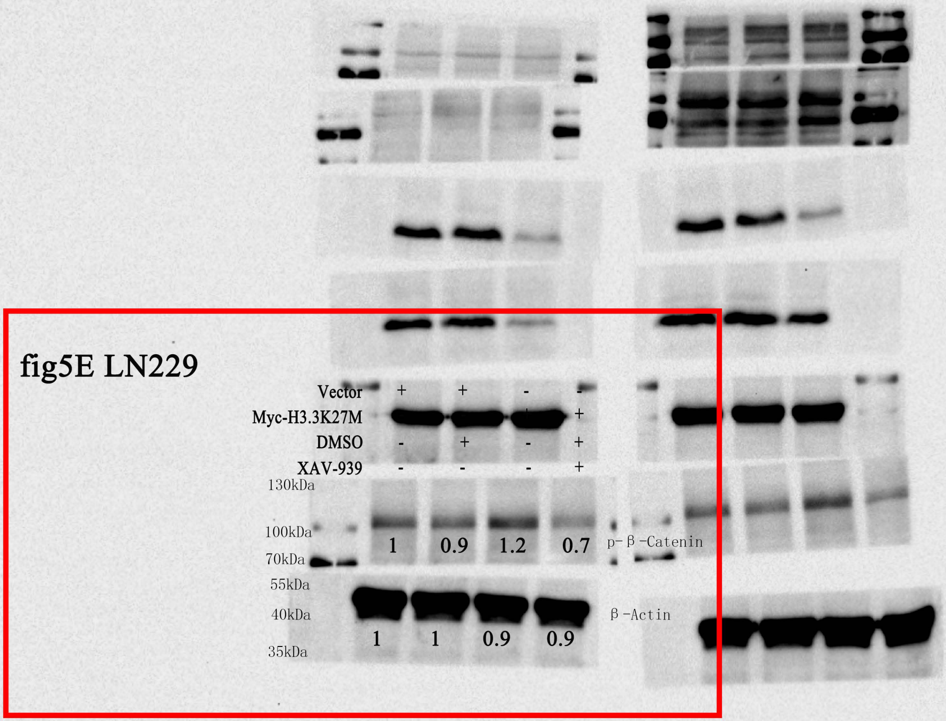
fig5E LN229
Vector + + - -
Myc-H3.3K27M + + + +
DMSO - + - +
XAV-939 - - - +
130kDa
100kDa
70kDa
55kDa
40kDa
35kDa
1 0.9 1.2 0.7
p-β-Catenin
β-Actin
1 1 0.9 0.9
