# Supplementary material for: H3F3A K27M Mutation Promotes the Infiltrative Growth of High-Grade Glioma in Adults by Activating β-Catenin/USP1 Signaling
Source: Cancers (Basel). 2022 Oct 3;14(19):4836. doi: 10.3390/cancers14194836 (PMC9563249; doi:10.3390/cancers14194836)
Supplement: Supplementary file 1 [file cancers-14-04836-s001.zip › cancers-1886589-supplementary.pdf]

Table S1. Patient Demographics.

Serial number	Grade	Subtype	Location	Gender	Age
N1	Non tumor		Right frontal lobe	male	26
N2	Non tumor		Left frontal lobe	male	53
N3	Non tumor		Right temporosphenoid lobe	male	55
N4	Non tumor		Left frontal lobe	male	40
N5	Non tumor		Right temporosphenoid lobe	female	65
N6	Non tumor		Right frontal lobe	female	44
T1	Grade II	Oligodendroglioma	Right frontal lobe	male	27
T2	Grade II	Oligodendroglioma	Right frontal lobe	male	41
T3	Grade II	Diffuse astrocytoma	Right frontal lobe	female	39
T4	Grade II	Oligodendroglioma	Left frontal lobe	male	31
T5	Grade II	Diffuse astrocytoma	Right temporosphenoid lobe	male	43
T6	Grade II	Diffuse astrocytoma	Right frontal lobe	male	38
T7	Grade II	Diffuse astrocytoma	Right temporosphenoid lobe	male	40
T8	Grade III	Anaplastic astrocytoma	Left frontal lobe	female	66
T9	Grade III	Anaplastic oligodendroglioma	Right temporosphenoid lobe	male	48
T10	Grade III	Anaplastic oligodendroglioma	Left frontal lobe	female	59
T11	Grade III	Anaplastic oligodendroglioma	Right frontal lobe	male	53
T12	Grade III	Anaplastic oligodendroglioma	Fourth ventricle	female	19
T13	Grade III	Anaplastic oligodendroglioma	Left temporosphenoid lobe	female	55
T14	Grade III	Anaplastic oligodendroglioma	Right temporosphenoid lobe	male	76
T15	Grade IV	Glioblastoma	Right frontal lobe	female	37
T16	Grade IV	Glioblastoma	Brainstem	female	54
T17	Grade IV	Glioblastoma	Left temporosphenoid lobe	female	65
T18	Grade IV	Glioblastoma	Thalamus	male	43
T19	Grade IV	Glioblastoma	Left frontal lobe	male	30
T20	Grade IV	Glioblastoma	Right frontal lobe	female	53
T21	Grade IV	Glioblastoma	Right temporosphenoid lobe	male	73
T22	Grade IV	Glioblastoma	Right frontal lobe	female	57
